# Effect of Tamarillo Fortification and Fermentation Process on Physicochemical Properties and Nutrient and Volatiles Content of Yoghurt

**DOI:** 10.3390/foods11010079

**Published:** 2021-12-29

**Authors:** Tung Thanh Diep, Michelle Ji Yeon Yoo, Elaine Rush

**Affiliations:** 1 School of Science, Faculty of Health and Environment Sciences, Auckland University of Technology, Private Bag 92006, Auckland 1142, New Zealand; tung.diep@aut.ac.nz; 2 Riddet Institute, Centre of Research Excellence, Massey University, Private Bag 11 222, Palmerston North 4442, New Zealand; elaine.rush@aut.ac.nz; 3 School of Sport and Recreation, Faculty of Health and Environment Sciences, Auckland University of Technology, Private Bag 92006, Auckland 1142, New Zealand

**Keywords:** tamarillo, yoghurt, fermentation, physicochemical properties, vitamins, volatiles

## Abstract

Bright-red Laird’s Large tamarillo is a unique and under-utilised fruit that is a dietary source of carotenoids, vitamins C and E, and dietary fibre. The effects of the addition of freeze-dried tamarillo powder (5–15%) to milk and yoghurt starter either before (PRE) or after (POS) fermentation on physicochemical properties were examined. Using LC-MS and GG-MS, nutrient and volatile contents of tamarillo yoghurt were also examined. The addition of tamarillo prior to fermentation was associated with a more yellow colour and higher concentrations of tocopherol compared to when tamarillo was added after fermentation. Higher elastic modulus, PUFAs, pro-vitamin A content, and vitamin C retention were observed for POS than PRE. All tamarillo yoghurts showed improvement in syneresis, lower lactose content, and higher concentrations of antioxidant vitamins than the commercial premium-assorted fruits yoghurt from New Zealand Food Composition Data. Yoghurt fortified with tamarillo powder offers the potential for the development of a high-value nutritional product that could be a good source of vitamin C and a source of vitamin E and β-carotene, and maintain the volatiles that give tamarillo its distinctive flavour.

## 1. Introduction

Tamarillos (*Solanum betaceum* Cav.) are a dietary source of polyphenols, including anthocyanins (delphinidin rutinoside, pelargonidin rutinoside, and cyanidin rutinoside), hydroxy benzoic acids (gallic acid), hydroxycinnamic acids (chlorogenic acid, caffeic acid), flavonols (kaempferol), flavanols (catechin, epicatechin), and flavonol glycosides (rutin, kaempferol-3-rutinoside); fibre; carotenoids (β-carotene); potassium; and vitamins C, E, and B6 [1,2,3]. Many of these constituents are strong antioxidants that are associated with health benefits such as reducing lipid oxidation and reducing risk for certain cancers, cardiovascular disease, and type 2 diabetes mellitus [4,5]. Tamarillo fruit has the potential to be a functional food or a functional ingredient for food reformulation. 

Recently, the formulation of functional fermented dairy products through the addition of fruit powder has become popular [6]. Yoghurt is already known to possess positive health benefits from the presence of probiotics. It is a good source of proteins, calcium, potassium, phosphorus, and vitamins [7]. Compared to other dairy products, yoghurt is gaining more popularity due to the presence of probiotics, nutrients in higher digested form, gel-like structure, taste, and mouthfeel [8]. It also has protective capacity against pathogenic bacteria, viruses, and intestinal infections that often lead to diarrhoea. The protective capacity comes from either the acidity of yoghurt or the inhibitor molecules generated by fermentation and specific starter cultures [7]. Many researchers have supplemented yoghurt with different fruit juices, pulp, seeds, and pomace and investigated changes in textural and rheological properties as well as antioxidant activity associated with the addition of fruit-derived bioactive compounds [8,9,10,11]. Yoghurts also provide an ideal environment to carry bioactive phytochemicals to the human body. The bioactive compounds can be added prior to fermentation as an ingredient, or after fermentation as part of the usual practice of adding flavouring and colouring agents [12]. In our previous work, we showed that freeze-dried tamarillo powder contains 10.6 mg/100 g α-tocopherol, 3.4 mg/100 g β-carotene, and 162 mg/100 g vitamin C, demonstrating the unique properties of NZ-grown Laird’s Large tamarillo [2]. Tamarillo fruits have not been used as ingredients in yoghurt production before. In this study, the effects of the addition of freeze-dried tamarillo powder (5–15%) to milk and yoghurt starter either before or after fermentation on physicochemical, rheological, and textural properties and the nutrient, volatiles, and fatty acid content of tamarillo yoghurt were examined. It was hypothesised that the addition of the powder before fermentation would enhance bioactivity, but after fermentation would preserve the attractive red colour of the tamarillo. 

## 2. Materials and Methods

### 2.1. Materials

Tamarillo (Laird’s Large cultivar), standard milk (Anchor blue top) purchased from a local supermarket (Auckland, New Zealand), and starter culture (*Lactobacillus delbrueckii subsp. Bulgaricus* and *Streptococcus thermophilus* (YoFlex^®^ Express 1.1 powder) from CHR Hansen (Hoersholm, Denmark)) were used as yoghurt ingredients.

The milk comprised 3.3 g protein, 3.4 g total fat, 4.8 g carbohydrates, 40 mg sodium, 117 mg calcium, 43 μg vitamin A, and 0.2 mg vitamin B_2_ per 100 mL. Tamarillo fruits were washed thoroughly with tap water and dried, and then the skin was peeled. Pulp was freeze-dried (Alpha 1–2 LD plus Freeze Dryer, Martin Christ, New Zealand) and ground to powder.

All chemicals and reagents used were AnalaR grade or purer. A mixture of 37 fatty acid methyl ester (FAME) standards (Supelco 47885-U), as well as analytical standards of reducing sugars, organic acids, α-tocopherol, β-carotene, and ascorbic acid, were purchased from Sigma-Aldrich (Auckland, New Zealand). Milli-Q water was produced by a Purite Fusion Milli-Q water-purifying machine (Purite Limited, Thame, Oxon, UK).

### 2.2. Yoghurt Preparation

Yoghurt samples were prepared using commercial yoghurt makers (Davis & Waddell, Steven, New Zealand). For the control yoghurt, starter culture and milk in the ratio of 0.1:100 (*w/w*), were placed in the yoghurt maker and set at 45 °C for 8 h and until the pH dropped below 5.0. The yoghurt was stored at 4 °C overnight and then homogenized at 4000 RPM (L4R Laboratory Mixer, Silverson, Waterside, UK) for 2 min [13]. Then, the yoghurt was divided into two parts. The first part was stored at 4 °C for analyses of physical properties, some proximate compositions, rheology, texture, and microstructure. The second part was snap frozen by liquid nitrogen, lyophilised, and ground to powder. The freeze-dried yoghurt powder was stored in a freezer at −20 °C for analyses of reducing sugars, organic acids, and antioxidant vitamins.

Freeze-dried tamarillo pulp powder (5, 10 and 15%) was added to the yoghurt either before (PRE) or after (POS) the fermentation process. For PRE, tamarillo powder was added to the mixture of milk and starter culture at the start of the yoghurt-making process, prior to fermentation. For POS, tamarillo powder was added to yoghurt in the final homogenization step. Seven preparations in total were prepared.

A preliminary experiment was conducted to determine the suitable level of fortification of yoghurt for appropriate texture and flavour, at concentrations of 1 to 20%. A difference in flavour to the control could not be detected when fortification was less than 5%. Fortification of above 15% was associated with an undesirably thick texture. Therefore, 5, 10, and 15% fortification were chosen for further experiments.

### 2.3. Physicochemical Property Analysis of Yoghurt Samples

Moisture, ash, protein, fat, carbohydrates, and dietary fibre contents were determined according to the AOAC methods [14]. Total soluble solids (TSS) were identified using a Rudolph refractometer (J57 Automatic Refractometer, Rudolph Research Analytical, Hackettstown, USA). Colour was recorded using a Hunter Lab (45/0, Colorflex EZ) colour analyser in L*, a*, and b* values. The pH was measured with a digital pH meter (HI 207, Hanna Instruments, Smithfield, RI, USA) equipped with a glass electrode without dilution of samples. Titratable acidity was determined based on AOAC method 942.15 [14], with dilution in MilliQ water (in 1:4 (*v/v*) ratio) followed by titration against 0.1 M NaOH until the pH reached 8.1. The results are expressed as a percentage of lactic acid.

The syneresis index of the yoghurt was determined according to Wang et al. [13] with some modifications. Twenty grams of yoghurt sample were centrifuged (Eppendorf Centrifuge 5804R, Medi’Ray, Auckland, New Zealand) at 1500 RPM for 10 min at 4 °C using an Eppendorf centrifuge (ThermoFisher Scientific, Braunschweig, Germany). The clear supernatant was poured off, weighed, and recorded as syneresis (%), which was calculated as Equation (1):

(1) $$\mathrm{Syneresis}\;\left(\%\right)=\frac{W_{f}}{W_{i}}\times 100$$

where W_i_ = weight of initial sample and W_f_ = weight of supernatant.

### 2.4. Rheological and Textural Analysis

Some modifications were made from Kristo et al. [15] for rheological measurements using a rheometer (RST-SST, Brookfield Ametek, Middleboro, MA, USA). The rheometer was calibrated following the manufacturer’s instructions before these measurements. For rheology, viscosity standard fluid (Brookfield Ametek) was used for calibration at 25 °C. For texture, the calibration was carried out using force with an appropriate calibration weight (50 g) on the calibration platform. To determine the viscosity profile (viscosity, consistency coefficient, flow behaviour index), a concentric cylinder geometry (diameters of the cup and bob were 28.92 and 26.66 mm, respectively) was used. A 20 mL sample of yoghurt was transferred into the cup, and a trap was used to prevent evaporation. Steady-state viscosity measurements were performed at shear rates from 1 to 400 s^−1^. To determine the elastic modulus, a vane spindle (SSVANE-) was used and the measurement was conducted at a speed of 0.5 rpm for 5 min. Data were acquired and processed with Rheo 3000 software.

A backward-extrusion test was conducted in yoghurt samples using a TA-XT plus texture analyser (Texture Technologies Corp, New York, NY, USA) according to Wang et al. [13] with some modifications. The following parameters were applied: cylinder probe diameter of 50 mm, test speed of 1.0 mm/s, penetration distance of 25 mm, and surface trigger force of 10 g. Firmness (N), consistency (N*s), and cohesiveness (N) were calculated from the exponent software. The test was carried out at ambient temperature with a sample temperature of approximately 4 °C. The samples were taken directly from the refrigerator at 4 °C and placed on the instrument stage for measurements.

### 2.5. Volatile Analysis

A 1–2 g yoghurt sample was quickly introduced into a 10 mL headspace vial. Ten μL of internal standard (6.5 mg/L solution of dichlorobenzene in water) was added to the vial and vortexed for 30 s. GC-MS coupled with SPME fibre was used for volatile analysis according to our previous study [16]. The total run time was 37 min. 

### 2.6. Fatty Acid Analysis

Fatty acids were analysed according to Sun and Zhao [17] with some modifications. A serial FAME standard solution was prepared in hexane and biphenyl solution (200 μg/mL:10 mg biphenyl in 50 mL chloroform) and used as internal standard. Approximately 0.5 g of the yoghurt sample was quickly introduced into a centrifuge tube. Ten mL of hexane and 1 mL of sodium methoxide (5.4 M) in methanol solution were added to the vial. After strong vortexing for 2 min and centrifuging at 3000 RPM for 10 min, 0.45 mL of the clear hexane layer was transferred to an autosampler vial and 50 μL of internal standard was added before injecting into GC-MS. 

The GC-MS and DB- FATWAX-Ultra Inert (UI) column (Agilent Technologies, Victoria,, Australia), measuring 30 m × 250 μm × 0.25 μm, was used to analyse the fatty acids. Chromatographic conditions were as follows: The oven was held at 50 °C for 2 min, then raised to 200 °C at a rate of 20 °C/min and then raised to 240 °C at a rate of 5 °C/min and held for 10 min. The equilibration time and total run time were 0.5 and 27.5 min, respectively. The inlet conditions were as follows: Helium was the carrier gas. The mode of injection was split, and the inlet temperature for the injection port was set to 250 °C. The injection volume was 1 μL and the spilt ratio was 10:1. The MS was operated in the SIM mode with a source temperature of 250 °C, a quadrupole temperature of 150 °C, and a transfer line temperature of 250 °C. Concentrations of 35 fatty acids were quantified using Agilent MassHunter Quantitative software (Agilent Technologies, Mulgrave, Australia). Peak areas were calculated for the reference ion for each target in each sample, standard, and blank, and normalised to recovery of the internal standard. Standard curves were constructed using linear relationships between relative peak area and concentration. Quantification of fatty acids in tamarillo yoghurt was implemented using standard calibration curves fitted with at least six suitable concentrations. A coefficient of correlation (r^2^) of >0.99 was obtained for all fatty acids.

The lipid indices of the yoghurt, including atherogenic index (AI), thrombogenic index (TI), and saturation index (SI), were calculated as Equations (2)–(4) [18]:

AI = (C12:0 + 4 * C14:0 + C16:0)/(Σ MUFAs + Σ PUFAs)(2)

TI = (C14:0 + C16:0 + C18:0)/[(0.5 * Σ MUFAs) + (0.5 * Σ n − 6) + (3 * Σ n − 3) + (Σ n − 3/Σ n − 6)](3)

SI = (C14:0 + C16:0 + C18:0)/(Σ MUFAs + Σ PUFAs)(4)

where Σ MUFAs is the sum of monounsaturated fatty acids and Σ PUFAs is the sum of polyunsaturated fatty acids.

### 2.7. Reducing Sugar and Organic Acid Analysis

Identification and quantification of reducing sugars were implemented according to our previous study [3]. Analytical standards of fructose, lactose, galactose, glucose, mannose, ribose, rhamnose, maltose, arabinose, xylose, fucose, and glucuronic acid were used for calibration. The profile of organic acid was identified based on Trigueros et al. [19] with some modifications and then quantified using LC-ESI-MS/MS. Briefly, freeze-dried samples (100 mg) were homogenized in 1 mL ultrapure water acidified with 0.1% formic acid and shaken vigorously for 30 s. The samples were incubated for 30 min at 4 °C and then centrifuged for 10 min at 10,000 rpm at 4 °C. The supernatants and the blank (0.1% formic acid) were injected into an LC-MS/MS system. Separation was performed using the Kinetex C18 column (100 × 2.1 mm, 1.7 μm; Phenomenex, Torrance, CA, USA). Mobile phase A was 0.1% formic acid in Milli-Q and mobile phase B was 0.1% formic acid in acetonitrile. The LC gradient was set at 0–3 min, 100% of A; 3–5 min, 80% of A; 5–10 min, 50% of A; and 10–25 min, 100% of A. The flow rate and injection volume were 0.2 mL/min and 1 μL, respectively. The MS was run in the negative mode with a total time of 25 min. The MMI source operating at ESI parameters were a gas temperature of 300 °C and gas flow of 10 L/min. The nebulizer, capillary voltage, column temperature, and vaporizer temperature were 40 psi, 4 kV, 25 °C, and 200 °C, respectively. External standards used include malic acid, lactic acid, malonic acid, citric acid, succinic acid, fumaric acid, itaconic acid, vanillic acid, syringic acid, and trans-cinnamic acid. Quantification of these analytes was undertaken using external pure standard calibration curves fitted with at least six suitable concentrations. A coefficient of correlation (r^2^) of >0.99 was obtained for all reducing sugars and organic acids.

### 2.8. Analysis of α-Tocopherol, β-Carotene, and Ascorbic Acid

Identification and quantification of α-tocopherol, β-carotene, and ascorbic acid were implemented according to our previous study [2] without further modification. 

### 2.9. Statistical Analysis

Mean and standard deviation were calculated based on at least three independent measurements (*n* ≥ 3) for each experiment. Correlation was determined by the Pearson correlation coefficient, r. One-way analysis of variance (ANOVA) and Fisher’s (LSD) multiple-comparison tests were applied to identify whether significant differences existed among different yoghurts. Data analysis was carried out using SPSS 25.0 (IBM Corp., Armonk, New York, NY, USA) and the statistical significance level was set at *p* < 0.05. Principal component analysis (PCA) was used to assess variability among yoghurt samples, and the concentrations of various compositions were visualised by heatmap and hierarchical clustering. Both PCA and the heatmap were carried out using the MetaboAnalyst web interface [20].

## 3. Results and Discussion

### 3.1. Physical Properties and Proximate Compositions of Tamarillo Yoghurts

For colour, lightness (L*) decreased significantly (*p* < 0.05) by 1.1–2.1 times, whereas red/green (a*) increased as a higher dose of tamarillo powder was added. Fermentation process may have affected the colour results from the absorption of water by dietary fibre and polyphenols in tamarillo. Variation in the a* and b* value was significant (*p* < 0.05), as tamarillo powder has high redness compared to milk. POS showed more redness compared to the PRE of the same % fortification, e.g., by three times in POS5 compared to PRE5. This might have been due to a change in the acidity of the yoghurt during fermentation, causing decolouration of natural pigments such as anthocyanins in the yoghurt matrix [21]. The fermentation process might have favoured the release of some pigments from the tamarillo, mainly carotenoids, making the product more yellow. Therefore, most PRE samples showed a higher b* value than the POS ones, e.g., 2.5 times higher in PRE5 compared to POS5.

Fortification, regardless of POS or PRE, substantially reduced syneresis (Table 1). The addition of 10% and 15% tamarillo powder reduced the syneresis of yoghurt by approximately a half and one-third of that of the control, respectively (Table 1). Syneresis is the separation of liquid that occurs in weak gel-like structures such as yoghurt, and it is a visible defect with a negative influence on consumer acceptability. It is mainly related to rearrangement of protein molecules (or aggregation) caused by the difference in density between phases where whey proteins accumulate on the surface of yoghurt and expel the serum out of the food matrix [13]. PRE possibly marginally lowered the syneresis through interactions of the added fibres and polyphenols from tamarillo with milk during fermentation, which could have formed a stable gel structure. Syneresis was reduced with increased concentration of tamarillo (Table 1). A 5% increase in concentration was associated with a substantial 7% decrease in syneresis (from 16 to 9%). This phenomenon can be explained by insoluble fibre (cell wall in tamarillo) absorbing water through porous fibrillar structures [22] and enhancing the water-holding capacity of the yoghurt. In addition, soluble fibre, such as pectin, creates linkages with both the aqueous phase and protein in the gel to increase the gel strength. Pectin can further stabilise casein aggregates through electrostatic and steric stabilisation, which may lead to the formation of casein–pectin complexes [23]. Tamarillo contains a high amount of methoxyl pectin, which may form gel in an acidic environment, incorporating the free serum and reducing syneresis [24]. 

For both PRE and POS addition of tamarillo, TSS, ash, protein, carbohydrate, and dietary fibre contents were increased proportionally with the level of fortification, as shown in Table 1. In both of PRE and POS with 15% fortification, increases in TSS of 25–28%, ash of 22–24%, protein of 42–46%, and carbohydrates of 71–73% were observed. Fortification reduced fat content by 3–11% and syneresis by 63–68% compared to the control. Dietary fibre is often absent in yoghurt and dairy products. The addition of dietary fibre by fortification with tamarillo is able to change solubility, viscosity, hydration properties, oil-binding capacity, and antioxidant activity [25], and enhance the viability of probiotic bacteria and their colonization ability [26]. POS10, POS15, PRE10, and PRE15 showed higher dietary fibre content (0.7, 1.1, 0.7, 1.0%) compared to the 0.4% reported for premium-assorted fruit yoghurt found in New Zealand Food Composition Database (NZ-FCD) [27]. A standard serving of yoghurt is 150 g, and the daily recommended dietary intake for an adult is 30 g. 

Higher TSS, ash, and protein were found in POS (2.5–4.0%, 2.9–4.9%, and 6.7–9.9%, respectively) than PRE, but fibre content was not different. PRE samples that showed a lower Brix value than the POS of the same % fortification as sugars in tamarillo were converted into acid during the fermentation process. This caused higher acidity (lower pH) in PRE samples. Because the gel structure of yoghurt is mainly formed by the denaturation of milk proteins (κ-casein and whey protein) at low pH, controlling the acid formation during fermentation is very important. The control samples showed the highest pH value and the lowest acidity (>3–10% and <13–32%, respectively) compared to the tamarillo-fortified yoghurts. The presence of acids such as ascorbic acid, citric acid, and malic acid in tamarillo powder has contributed to further acidification [28]. A similar effect was also reported in yoghurt fortified with passion fruit peel powder [28].

### 3.2. Rheological and Textural Analysis

The control yoghurt had a negligible viscosity constant and 5% PRE and POS were also low. Both 10% and 15% were substantially more viscous. The increase between 5% and 10% was ~7×. The difference between 10% and 15% was 6× for PRE and 5× for POS (Table 2). The Oswald–de Waele power law model was used to model the flow behaviour of the fortified yoghurt. For all yoghurt samples, the correlation coefficient for the model fit was above 0.96 (data not shown). The flow behaviour index (n) of all samples was smaller than 1; hence, the yoghurt of all treatments in this study can be considered a non-Newtonian fluid with shear-thinning behaviour. Similar results were observed in yoghurt fortified with apple pomace [13] and orange pomace [29]. According to Cui et al. [30], the pseudoplastic behaviour of yoghurt was due to the breakage of bonds between the protein aggregates as a consequence of shear stress. The increase in tamarillo powder concentration led to the increase in the consistency coefficient index and the decrease in the flow behaviour index. On the other hand, the fermentation process did not significantly affect those two values. The water-holding capacity of tamarillo powder might have attributed to the increase in the consistency coefficient value.

The elastic modulus describes the resistance of a material to deformation and it relates to the solid-like characteristic that arises from the internal network structure. As shown in Table 2, the increase in the tamarillo ratio resulted in a higher elastic modulus of fortified yoghurt samples with a significant difference (*p* < 0.05 for trend). The higher elastic modulus of 15% tamarillo yoghurt is indicative of a structure that tended more towards solid than liquid. PRE resulted in a slightly lower elastic modulus compared to POS. The type of interaction occurring between the fibres and polyphenols of tamarillo (mainly) and milk protein could contribute to this parameter. According to Chetachukwu et al. [31], this interaction can either be segregative or associative, depending on three different phenomena (co-solubility, complexation, and incompatibility). The hydration of fibres may also enhance the protein–protein interaction, resulting in the increased gelation of the yoghurt structure (complexation). Apple, bamboo inulin, and wheat as source of dietary fibre have been used to improve the rheological properties of yoghurt [32]. The range of these interactions is governed by directed hydrogen-bonding interactions that extend further than the electrostatic-dependent screening length. It is also possible that the already weak protein structure of milk may have been disrupted. This is because some heat-labile milk protein molecule may have undergone denaturation during fermentation, thereby limiting its surfactant capability at the oil–water interface of the emulsion.

According to Walia et al. [33], the texture parameters are the key indicators for evaluating the physical and sensory quality of dairy products. These parameters depend not only on the arrangement of molecules in the gel network of products but also on other factors such as manufacturing processes, protein and fat composition, other added ingredients, and particularly the concentration of properties of fibres. The addition of tamarillo powder significantly (*p* < 0.05) improved the firmness, consistency, and cohesiveness of the yoghurt, as the dose of tamarillo powder increased regardless of the fermentation process (Table 2). This may be explained by the phenolics and anthocyanin compounds present in tamarillo, which have hydroxyl (−OH) groups with a strong affinity for proline-rich proteins like casein [34]. Similar findings with pomegranate extract were observed by Pan et al. [35].

Fortification at 15% and PRE doubled the firmness and consistency compared to the control (Table 2). According to Paseephol et al. [36], the firmness of yoghurt depends on the total solid content of the milk mixture. Several factors affect the firmness of fermented milk, such as the type of protein, the interaction between the ingredients used, and the composition of the starter culture [37]. By adding in dry tamarillo powder, total solid content also increased, and this may explain the higher firmness compared to the control. At 5 and 10% fortification, both POS and PRE samples showed similar firmness. However, for 15% fortification, PRE showed a higher increase in firmness of about 17% than POS. The time of interaction (8 h for fermentation) allowed more binding sites between milk protein and tamarillo components (mainly amino acid side chains and polyphenol aromatic rings) to be produced, and could explain the firmer texture. *L. delbrueckii* ssp. *bulgaricus* may also have contributed to this increase via exopolysaccharides, as shown in another study [38].

According to Yildiz and Ozcan [39], high consistency refers to a viscous product with high density. POS5 and PRE5 showed no change in consistency from the control. With 10 and 15% fortification, significantly higher consistency (*p* < 0.05) was observed compared to the control (1.3–1.4 and 1.5–2.0 times, respectively). POS10 and PRE10 were similar in consistency, but by contrast, at 15% fortification, PRE produced higher consistency than POS. This may be explained by formation of complexes between fibre and polyphenols from tamarillo, with the protein aggregates as part of the structural network formation, as observed by others [35].

According to Peng et al. [40], the higher cohesiveness values with the addition of protein allows for the breakage of a large number of casein–casein and protein linkages during stress application to reform after the stress is released. Cohesiveness is directly related to the internal strength of the material structure. All of the fortified samples showed significantly higher cohesiveness than the control (*p* < 0.05) (Table 2) and the protein concentration of 15% PRE and POS was almost double that of the control (Table 1). Change in consistency did not show a clear pattern between POS and PRE across different levels of fortification. The increase in cohesiveness of the fortified samples may be attributable to the increase in viscosity from the addition of tamarillo powder particles (both soluble and insoluble compounds), reinforcing the internal gel structure. It is likely that the rehydrated tamarillo powder absorbed the separated whey and also trapped casein clusters generated from shearing, which may have strengthened the loose and opened protein structure to improve the consistency.

### 3.3. Volatile Profile

A total of 85 and 107 volatile compounds were identified in the control and fortified yoghurt samples, respectively. These were further classified into 22 alcohols, 19 esters, 15 acids, 13 benzenes, 9 aldehydes, 9 ketones, 5 hydrocarbons, 4 furans, 3 nitrogen compounds, 3 sulphur compounds, 2 terpenes, and 2 pyrans, as seen in Supplementary Table S1. Ketones were the most abundant in the control, POS5, POS10, and PRE5, and esters dominated POS15, PRE10, and PRE15 (Supplementary Table S2). Among ketones, acetone with a sweet and fruity aroma dominated in PRE samples, owing to the fermentation process. Fortification with tamarillo significantly (*p* < 0.05) influenced the presence of acetone as well. Acetone is usually present in small quantities in milk, and further production has been carried out by starter cultures through symbiotic activity [41,42]. The highest activity of the yoghurt cultures during the production process of acetone was “during milk coagulation and cooling, up to 7 h” with maximum concentration of 66.0–75.5 μg/100 g [42]. Acetoin, which has a creamy, slightly sweet and butter-like flavour [42], was abundant (10.84–28.62 μg/g yoghurt) in the control and POS samples. Acetoin is a common flavour compound in cultured dairy products and is converted from diacetyl by diacetyl reductase [41], reducing the harsh flavour [43].

The addition of tamarillo significantly increased (*p* < 0.05) methyl hexanoate and ethyl hexanoate in yoghurt samples (36–80 times) compared to the control. These esters mainly originate from tamarillo [44,45,46] and can minimize the sharpness and bitterness imparted by fatty acids and amines in yoghurt [41]. The concentration of ethyl hexanoate in fortified yoghurts was significantly lower than the original amount present in tamarillo fruit [16]. This may have been due to the matrix effect of yoghurt, where proteins and exopolysaccharides, as well as physicochemical interactions between components like pectin and sucrose, affect the release of volatile compounds [42]. The concentration of volatile compounds in the headspace decreased when the matrix changed from water (i.e., tamarillo fruit) to yoghurt.

Acidity, perceived as sourness, is crucial for the flavour acceptance of yoghurt [41]. Carboxylic acid was the second most dominant group in the volatile profile of most yoghurt samples, except for POS15 (Supplementary Table S2). Hexanoic acid and butanoic acid were the two most abundant carboxylic acids in all samples. The highest amounts of these acids were found in the control (18.3 and 13.3 μg/g yoghurt), and POS had 1.3–1.7 times higher concentrations compared to PRE of the same level of fortification. Acetic acid and octanoic acid were also present at substantial levels, which could have contributed to the vinegar/acid notes and fruity notes, respectively [41].

Acetaldehyde, 3-methyl-butanal, and 2-hexenal (E) were dominant aldehydes in tamarillo yoghurts that carried a fruity aroma. Acetaldehyde is formed from the breakdown of threonine by threonine aldolase, which is present in both *Lb. bulgaricus* and *S. thermophilus* [42]. Although the concentration of acetaldehyde was 1.04 μg/g yoghurt in the control, fortification dropped its concentration as low as 0.12 μg/g yoghurt, with a higher concentration seen in POS in general. Similarly, lower acetaldehyde concentration was observed in other studies with fruit fortification in yoghurt [42]. The addition of tamarillo resulted in extra volatile compounds that are normally not found in yoghurt, and including benzenes, hydrocarbons, nitrogen and sulphur compounds, terpenes, and pyrans.

**Figure 1:**
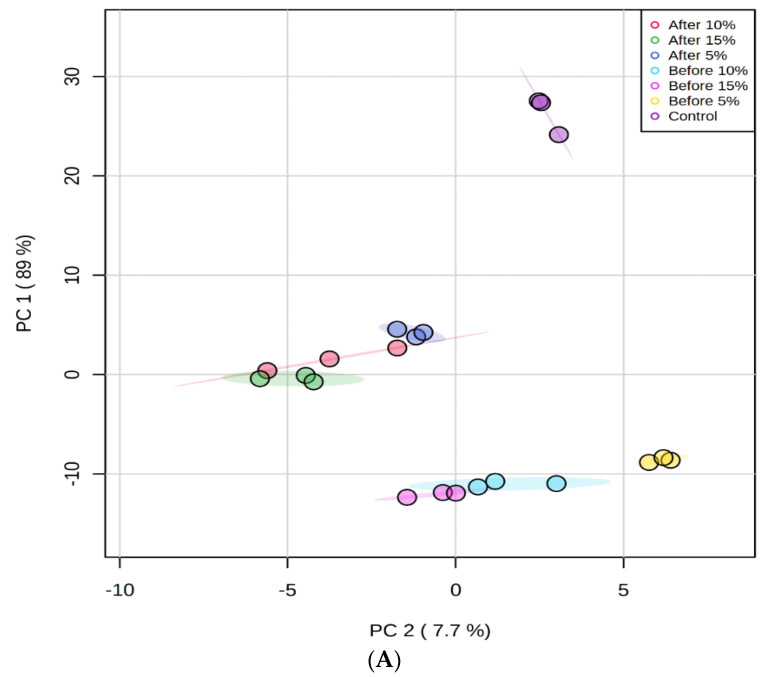

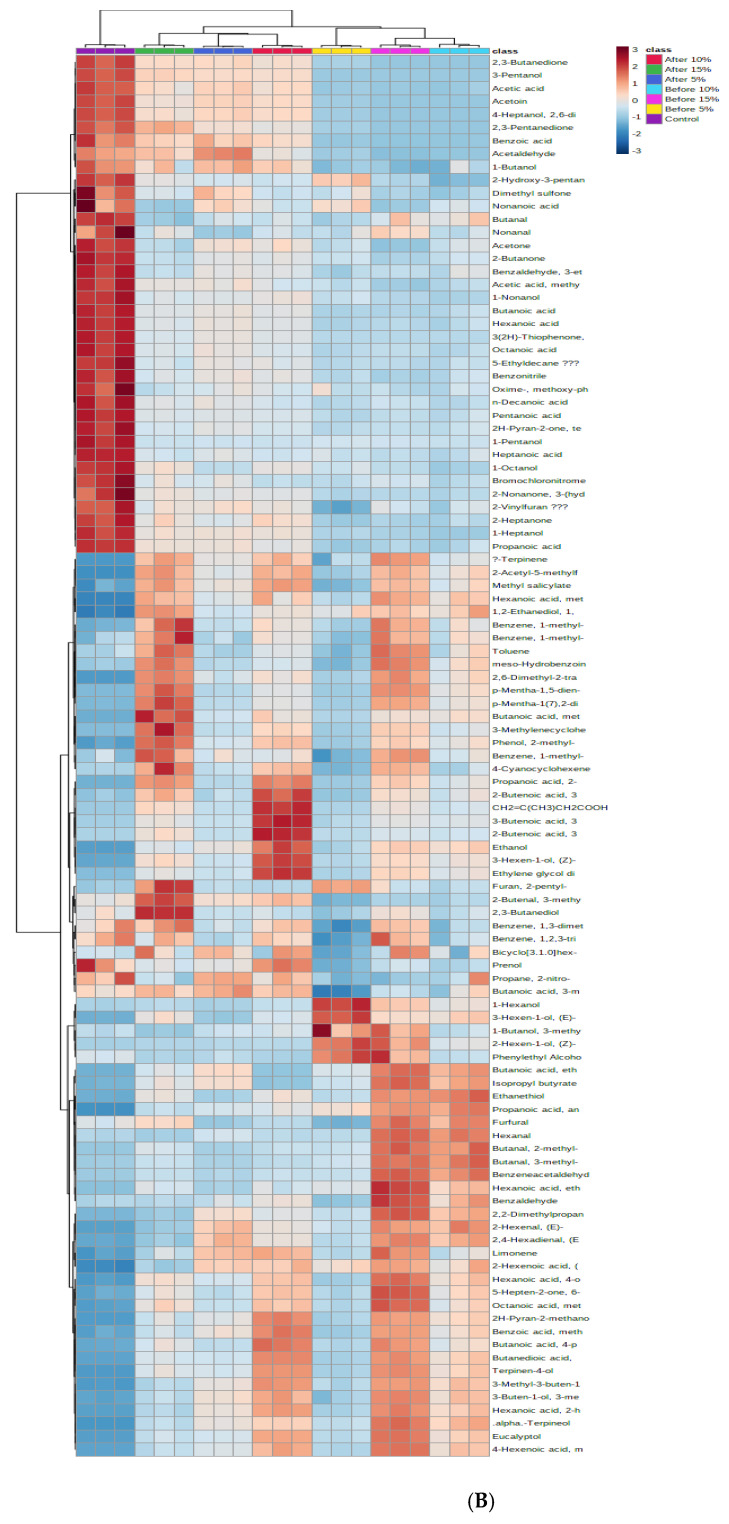
Score plot (**A**) and heatmap (**B**) diagrams from the principal component analysis of the relative concentration of volatiles for control yoghurt and tamarillo yoghurts produced from two fermentation processes. The vertical bar (3 to −3) from deep red to deep blue shows high to low levels of each volatile in different groups of yoghurt samples.

The effects of fortification were further analysed by principal component analysis (PCA) and presented as the PCA plot and heatmap in Figure 1A,B, respectively. The separation of control and tamarillo yoghurts based on the volatile profile was clearly visible. Principal component (PC) 1 and PC 2 explained 89% and 7.7% of the variance, respectively, and therefore, no further PCs were considered. For PC1, the control sample was located on the positive region, whereas all of POS were around the zero line and all of PRE fell in the negative region. For PC2, the control was located in the positive region, whereas all POS were located in the negative area. PRE, PRE5, and PRE10 were in the positive area but PRE15 fell in the negative region. PC2 completely resolved the fortified yoghurt samples based on the tamarillo concentration regardless of fermentation process. The 5% samples were on the far right side, the 15% samples were located on the left side, and the 10% samples fell in between the 5% and 15% samples. A heatmap was generated to visualise the relative abundance of volatiles in each yoghurt group. Overall, clear differences were observed between control, PRE, and POS yoghurt samples.

### 3.4. Fatty Acid Profile

The addition of tamarillo significantly (*p* < 0.05) increased UFA content and caused a subsequent decrease in SFAs to below 70% for both PRE and POS (Supplementary Table S3). In all yoghurt samples, palmitic acid (C16:0) was the most abundant SFA, as reported before in several other studies [18,47,48], followed by myristic acid (C14:0) and then stearic acid (C18:0) (Supplementary Table S3). The addition of tamarillo increased the PUFAs by 3.2–3.8, 5.3–6.2, and 6.5 times for 5, 10, and 15%, respectively, compared to the control for both PRE and POS. Oleic acid (C18:1) and linoleic acid (C18:2) dominated monounsaturated fatty acid (MUFA) and PUFA profiles, respectively. Compared to the control, an increase in oleic acid was observed in POS but a decrease was observed in PRE. The increase in PUFAs observed in fortified yoghurt may have been caused by the presence of PUFAs (account for 72.05%) [49] in the mucilage of tamarillo pulp used in the study. A synergistic interaction between fruit fibre and the probiotic strain may also have contributed to the increase as well [26]. PUFAs in PRE were lower than in POS, which may be explained by the continuous oxidization of PUFAs to hydroperoxides and subsequently aldehydes [41]. This finding also supports the elevated amount of aldehydes in PRE compared to the POS in Supplementary Table S2. POS5, POS10, POS15, and PRE15 showed similar or even higher C18:2 ω-6 content than the assorted fruits yoghurt (66 mg/100 g) reported in New Zealand Food Composition Database [27]. Total amounts of ω-3 and ω-6 increased significantly (*p* < 0.05) compared with the control, by 1.4–1.5 and 2.0–2.6 times higher in linolenic acid content for PRE and POS than the control, respectively. For linoleic acid, the increase was 5–8.5 and 7.9–13.7 times for PRE and POS compared to the control, respectively.

Atherogenic (AI), thrombogenic (TI), and saturation indices (SI) were calculated as a measure of nutritional quality indices [26,47]. The AI indicates the relationship between the sum of the main SFA and the sum of MUFAs plus PUFAs. The SFAs (C12:0, C14:0, and C16:0) are considered pro-atherogenic (favouring the adhesion of lipids to cells of the immunological and circulatory system). The MUFAs and PUFAs are considered antiatherogenic (inhibiting the aggregation of plaque and diminishing the levels of esterified fatty acid, cholesterol, and phospholipids, thereby preventing the appearance of micro-coronary and macro-coronary diseases) [18]. This value shows the correlation of the risk of atherosclerosis, i.e., the increase in the level of blood cholesterol with the increase in SFAs or the decrease in ΣMUFAs and ΣPUFAs. The TI value presents the tendency to form clots in the blood vessels, which is defined as the relationship between pro-thrombogenic (saturated) and anti-thrombogenicity acids (MUFAs, ω-6 PUFAs, and ω-3 PUFAs). According to Ribeiro et al. [18], the influence of the different FAs ingested on coronary heart disease have been measured through these two values. The lower the AI and TI values, the greater the protective potential for coronary artery disease. In this study, tamarillo yoghurt exhibited lower values of AI and TI than the control sample. The addition of 5, 10, and 15% tamarillo powder resulted in a reduction of AI of 23, 35–38, and 42%, respectively, regardless of the fermentation process. The TI values also decreased by 79–87, 61–73, and 52–59% by adding 5, 10, and 15% tamarillo powder to yoghurt, respectively. The saturation index (SI) is another good indicator of the nutritional value of dietary fat, indicating the relationship between the sum of SFAs (pro-thrombogenic) and UFAs (anti-thrombogenic), and food with a lower SI would be considered healthier [18]. Yoghurt fortified with tamarillo presented a lower SI than the control (Supplementary Table S3). Regardless of the fermentation process, the addition of 5, 10, and 15% tamarillo powder reduced the SI by 24–25, 36–39, and 42–44%, respectively. The higher the tamarillo fortification, the lower the AI, TI, and SI values, which could be used as an innovative strategy to increase the health appeal of high-fat yoghurts.

**Figure 2:**
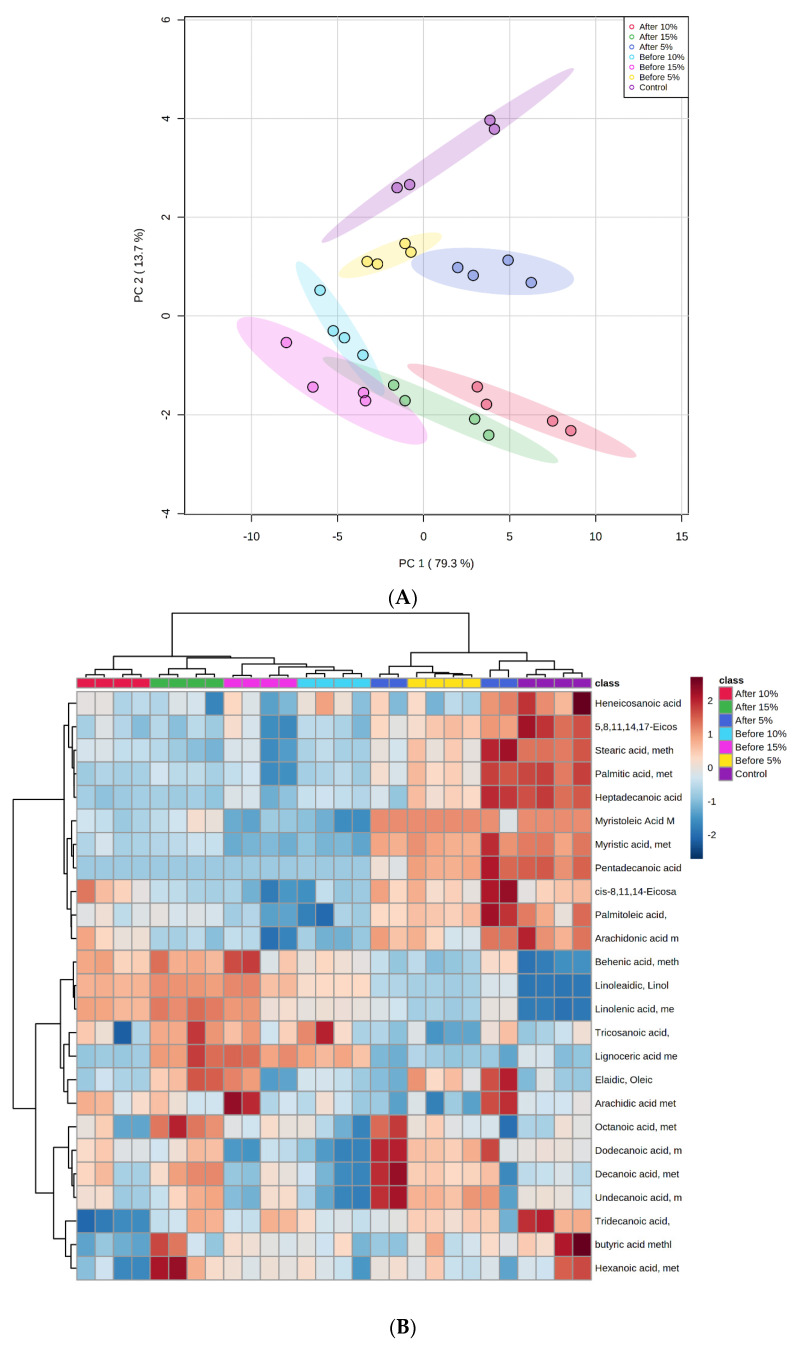
Score plot (**A**) and heatmap (**B**) diagrams from the principal component analysis of fatty acid contents for control yoghurt and tamarillo yoghurts produced from two fermentation processes. The vertical bar (2 to −2) from deep red to deep blue shows high to low levels of each fatty acid in different groups of yoghurt samples.

The PCA plot and heatmap were used to determine the effects of the addition of tamarillo powder, as shown in Figure 2A,B, respectively. The control and tamarillo yoghurt samples were almost perfectly resolved on PC1, which explained 79.3% of the variance in the data, and on PC2, which explained another 13.7% of the variance. Therefore, no further PCs were considered. PC1 mostly resolved the difference in tamarillo yoghurts between PRE and POS. For PC1, all of PRE fell in the negative region, whereas most of POS was located on the positive region, except for two samples of POS15. Two samples of the control yoghurt fell in the negative region and the other two were located in the opposite region. For PC2, the control, PRE5, and POS5 were located in the positive region. PRE10, POS10, PRE15, and POS15 were in the negative area, except for one sample of PRE10. The PC2 also completely resolved the samples based on the tamarillo concentration regardless of the fermentation process. The heatmap cluster analysis of these 37 fatty acids showed a clear separation between control and fortified yoghurts. Separation among tamarillo yoghurts based on the concentration of tamarillo added was also observed. The PRE5 and POS5 were more closely related to the control than the others. On the other side, PRE10 and PRE15 were in the same cluster, which was separated from the POS10 and POS15 cluster.

### 3.5. Reducing Sugar and Organic Acid Profile of Yoghurts

Lactose and maltose were the dominant sugars in all samples (Table 3), and the addition of tamarillo significantly increased (*p* < 0.05) the concentration of these sugars. A higher concentration of lactose was also found in tamarillo yoghurt produced from PRE than POS by 1.3–1.5 times. The concentration of lactose in cow milk with standard fat is 4.5 g/100 g [27]. During fermentation, the content of lactose in milk significantly decreases and converts to lactic acid [50]. This was observed in our results, with a lactose range of 1019–2277 mg/100 g, which was less than approximately 50% compared to the premium assorted fruits yoghurt (4100 mg/100 g) from the NZ-FCD [27]. 

As expected, with fruit added to the yoghurt the amount of fructose and glucose in fortified yoghurt (*p* < 0.05) was higher by 15–50 and 3–6 times, respectively, compared to the control [51,52]. Glucose content in all tamarillo yoghurts (214–495 mg/100 g) was lower than the fructose. The concentrations of fructose and glucose in all tamarillo yoghurts were lower than strawberry, peach, blueberry, raspberry, and mixed-fruit yoghurts, which ranged from 1200–4000 and 490–3890 mg/100 g, respectively, according to Rybicka and Gliszczyńska-Świgło [50]. Hence, the low levels of fructose and glucose and a relatively low ratio of fructose to glucose (1.7:1–2.5:1) in the fortified tamarillo yoghurt may be considerable for developing functional food. Comparing galactose content, an increase of approximately eight-fold from PRE to POS was observed. The cultures used in yoghurt fermentation (*L. bulgaricus* and *S. thermophilus*) utilise the galactose moiety of lactose rather than glucose. The concentrations of other sugars (ribose, rhamnose, mannose, arabinose, xylose, fucose) as well as glucuronic acid increased when yoghurt was fortified with tamarillo powder. 

Organic acids are important indicators of bacterial metabolic activity in yoghurt, and they are considered natural preservatives. Organic acids also contribute to the taste and flavour of the product, together with other volatile and semi-volatile compounds [53]. All organic acids detected were significantly (*p* < 0.05) affected by the type of yoghurt (Table 3). Lactic acid was the dominant organic acid in all samples, followed by citric acid, with significantly (*p* < 0.05) higher concentrations found in the fortified than the control yoghurt (*p* < 0.05). Citric acid is the predominant organic acid in milk as a product of bovine metabolism and it promotes fresh taste. This acid is known to be utilised during the fermentation process [54]. 

Malic acid possesses antimicrobial properties, where prevention of the growth of *Listeria monocytogenes*, *Salmonella gaminara*, and *Escherichia coli* O157 has been reported [55]. The high concentration of malic as well as citric acids in tamarillo yoghurt resulted from the use of tamarillo fruit, which is predominated by those two acids [56]. The concentration of malic acid increased with the increase in tamarillo concentration added. A similar phenomenon was also observed for cinnamic acid. Fumaric acid was only detected in yoghurt produced from POS15. This acid was found to be effective in the prevention of cardiovascular diseases [57]. Itaconic and syringic acids were only detected in tamarillo-enriched yoghurt. The itaconic concentration was not much different between PRE and POS, but increasing the fortification to 10 and 15% increased its content by 2.7–2.9 and 3.3–3.6 times, respectively. Itaconic acid has been shown to be a potential bacteriocide and is largely encountered as a component of the animal immune response [58]. The concentration of syringic acid in fortified yoghurts varied from 1.2 to 2.74 mg/100 g. POS showed slightly higher syringic acid concentration than PRE. This acid has been reported to have a wide range of therapeutic applications in the prevention of diabetes, cancer, and cerebral ischemia, and it also possesses antioxidant, antimicrobial, and anti-inflammatory properties. The strong antioxidant activity of syringic acid may confer beneficial effects for human health [59].

### 3.6. Determination of α-Tocopherol, β-Carotene, and Ascorbic Acid in Yoghurts

Adding tamarillo significantly increased α-tocopherol (vitamin E), β-carotene (pro vitamin A), and ascorbic acid (vitamin C) in yoghurt (Table 4). The highest α-tocopherol concentration was found in PRE15. This trend is a clear indication that tamarillo fortification caused a significant increase (*p* < 0.05) of approximately 5–11 times in vitamin E concentration in yoghurt regardless of the fermentation process. PRE10 and PRE15 possessed higher α-tocopherol content than their respective counterparts from POS. According to Sarkar et al. [60], freeze-drying, which reduced the water content, caused an increase in solid content, thus increasing the vitamin E concentration. Therefore, using tamarillo powder to fortify yoghurt should be more efficient than using fresh fruit or fruit juice. All the tamarillo yoghurt showed higher α-tocopherol content than the premium assorted fruits yoghurt (0.16 mg/100 g) from the NZ-FCD [27], although only POS15, PRE10, and PRE15 samples were considered a source of α-tocopherol. Tocopherols scavenge peroxide radicals by forming tocopheroxyl radical, and at higher temperature, by reacting with other antioxidants, this tocopheroxyl radical may be added back to tocopherol form [60]. This could explain the higher tocopherol in PRE than POS. Vitamin E has been reported to be an impressive antioxidant compound that can prevent lipid peroxidation and efficiently eliminate free radicals, and plays a key role in the prevention of several chronic diseases [60].

A significant amount of β-carotene was present in all types of yoghurt. Fortification significantly (*p* < 0.05) increased the β-carotene content by approximately 5–11 times (Table 4). Higher β-carotene content was observed in POS than in PRE. This might be because β-carotene was degraded due to the isomerisation process that took place during fermentation. Only the POS15 sample could be considered a dietary source of β-carotene (Table 4); however, other tamarillo yoghurt showed higher β-carotene content than premium assorted fruits yoghurt (0.07 mg/100 g) from the NZ-FCD [27]. The high content of β-carotene as well as α-tocopherol in tamarillo yoghurt compared to premium assorted fruits yoghurt resulted from the use of tamarillo fruit, which is rich in these antioxidant vitamins. Similar to the α-tocopherol, an increase in β-carotene content of freeze-dried fruit compared to fresh samples has been reported [51], which proves that fortification with tamarillo has potential. β-carotene has been reported to exhibit pro-oxidant and antioxidant activity against lipid peroxidation [60], which may affect the shelf life of yoghurt.

Tamarillo is a rich source of vitamin C and ascorbic acid [2], whereas in milk, ascorbic acid is present in low amounts, of 1.65–2.75 mg/100 g. Tamarillo possesses nine- to 15-fold higher vitamin C content than milk. Thus, tamarillo fortification increased the ascorbic acid content of yoghurt significantly (*p* < 0.05) (Table 4). The highest ascorbic acid content was in POS15. The direct addition of tamarillo powder without any further thermal treatment enabled a higher retention of vitamin C in fortified yoghurt in POS compared to PRE. All tamarillo yoghurt showed higher vitamin C content than the premium assorted fruits yoghurt (2.6 mg/100 g FW) reported in the NZ-FCD [27]. Most tamarillo yoghurts were a good source of vitamin C (Table 4), except for PRE5, and met a higher % of RDI than premium assorted fruits yoghurt (10%) [27]. The concentration of these three compounds in fortified yoghurt was lower than those present in raw fruit (α-tocopherol of 0.53 mg/100 g per 5% fortification, β-carotene of 0.17 mg/100 g per 5% fortification, and vitamin C of 8.12 mg/100 g per 5% fortification). Further interaction between the components in yoghurt and the matrix effect may have influenced the concentration of these analytes during extraction and analysis. Vitamin C is the most thermo-sensitive bioactive compound in plants and is susceptible to degradation from high temperature, moisture, and oxygen concentration [60]. This can explain the lower concentration of ascorbic acid in PRE compared to POS. Under aerobic conditions, ascorbic acid first converts to dehydro-ascorbic acid via a reversible reaction pathway and then further hydrolysis and oxidation takes place in an irreversible way [60]. Vitamin C has played a crucial role in human physiology and body metabolism. This compound can act as a strong antioxidant agent that can prevent lipid peroxidation and as a strong free radical scavenging agent that can remove different types of the harmful free radical present in the human body [60]. 

New Zealand is one of the leading producers and exporters of tamarillo; however, products produced from tamarillo remain scarce. Results from the current study show a potential for fortifying yoghurt with tamarillo, where improvements in flavour and nutritional properties were observed. Fortification with tamarillo may be applicable to other products where natural red colour from anthocyanins would be favoured or acceptable. The strengths of the current study are that this is the first study to fortify tamarillo powder into yoghurt either pre-fermentation or post-fermentation. Our results have demonstrated that tamarillo pulp powder can be utilised as added dietary fibre and antioxidant vitamins in yoghurt, where it can simultaneously impart desirable structural changes to the yoghurt matrix. In this case, tamarillo powder added to yoghurt mix before or after fermentation decreased syneresis substantially and increased the viscosity, firmness, consistency, and cohesiveness of the fortified yoghurts, which may be more appealing to the consumer. Tamarillo powder contributed a higher amount of dietary fibre and antioxidant vitamins to the yoghurt while retaining the fruit flavour. Considered a dietary source of pro-vitamin A (β-carotene), vitamin C, and vitamin E (α-tocopherol), tamarillo yoghurts may deliver health and nutritional benefits arising from the antioxidant vitamins. The total fat analysis was carried out by the Soxhlet technique, which may give inconsistent measures with dairy products. Analysis for minerals (iron, calcium, potassium) and other vitamins (B, D, K) in fortified yoghurt may give more information about the nutrient density of tamarillo yoghurt. A lack of sensory tests also caused insufficient understanding on how yoghurt flavour and texture would be perceived by the addition of fruit. We plan to study the interaction between fibres and stater culture (viability, total colony counts) in the future.

## 4. Conclusions

The addition of tamarillo in powder form both before and after fermentation increased the acidity, fibre, protein, and lactic acid contents of yoghurts and maintained the unique volatile compounds in the fruit. The addition of tamarillo resulted in a firmer yoghurt of lower deformability and higher elastic behaviour and viscosity. The addition of powder after fermentation preserved the red colour of the tamarillo and retained the vitamin C content. Yoghurt fortified with tamarillo powder offers the potential for the development of a value-added product that could be a good source of vitamin C and a source of vitamin E and β-carotene and maintain the volatiles that give tamarillo its distinctive flavour. Next steps include the determination of the organoleptic properties and consumer acceptability of tamarillo yoghurt.

## Figures and Tables

**Table 1:** Physical properties and proximate compositions of yoghurt samples and tamarillo powder.

Parameters	Tamarillo Powder (%/Dry Weight)	Control	POS5	POS10	POS15	PRE5	PRE10	PRE15
Lightness (L*)	-	76.53 ± 0.87 ^a^	52.98 ± 0.26 ^b^	40.07 ± 0.36 ^c^	35.23 ± 0.24 ^d^	69.8 ± 0.93 ^e^	50.05 ± 0.62 ^f^	39.92 ± 0.12 ^c^
Redness (a*)	-	−0.33 ± 0.10 ^a^	29.13 ± 0.23 ^b^	31.12 ± 0.25 ^c^	32.58 ± 0.39 ^d^	9.53 ± 0.40 ^e^	24.13 ± 0.28 ^f^	28.05 ± 0.19 ^g^
Yellowness (b*)	-	11.63 ± 0.43 ^a^	8.13 ± 0.29 ^b^	9.82 ± 0.10 ^c^	10.05 ± 0.15 ^c^	20.07 ± 0.56 ^d^	12.37 ± 0.23 ^e^	9.78 ± 0.13 ^c^
Syneresis (%)	-	30.19 ± 0.90 ^a^	22.88 ± 1.84 ^b^	18.21 ± 1.62 ^c^	11.18 ± 1.19 ^d^	20.65 ± 1.48 ^b^	15.78 ± 1.07 ^e^	9.59 ± 1.25 ^d^
pH	-	4.47 ± 0.03 ^a^	4.03 ± 0.01 ^b^	4.01 ± 0.02 ^c^	3.93 ± 0.01 ^d^	4.35 ± 0.00 ^e^	4.29 ± 0.02 ^f^	4.06 ± 0.01 ^g^
Acidity (%)	-	0.91 ± 0.02 ^a^	1.18 ± 0.04 ^b^	1.26 ± 0.03 ^c^	1.33 ± 0.03 ^d^	1.05 ± 0.02 ^e^	1.21 ± 0.03 ^b^	1.3 ± 0.01 ^d^
TSS (°Brix)	-	7.32 ± 0.19 ^a^	9.05 ± 0.14 ^b^	9.57 ± 0.08 ^c^	10.12 ± 0.13 ^d^	8.81 ± 0.06 ^e^	9.33 ± 0.06 ^f^	9.71 ± 0.07 ^c^
Moisture (%)	-	89.72 ± 0.02 ^a^	85.11 ± 0.05 ^b^	81.92 ± 0.08 ^c^	78.78 ± 0.01 ^d^	85.79 ± 0.16 ^e^	81.94 ± 0.11 ^c^	79.2 ± 0.13 ^f^
Ash (%)	8.26	0.53 ± 0.10 ^a^	0.61 ± 0.09 ^a^	0.67 ± 0.13 ^a^	0.7 ± 0.08 ^a^	0.58 ± 0.11 ^a^	0.64 ± 0.17 ^a^	0.68 ± 0.12 ^a^
Protein (%)	9.69	1.84 ± 0.08 ^a^	2.43 ± 0.11 ^b^	3.06 ± 0.05 ^c^	3.41 ± 0.08 ^d^	2.19 ± 0.10 ^e^	2.76 ± 0.07 ^f^	3.18 ± 0.09 ^c^
Fat (%)	2.36	2.93 ± 0.18 ^a^	1.54 ± 0.15 ^b^	1.92 ± 0.34 ^bc^	2.59 ± 0.22 ^ad^	1.72 ± 0.25 ^b^	2.28 ± 0.27 ^cd^	2.85 ± 0.21 ^a^
Carbohydrate (%)	31.7	2.37 ± 0.16 ^a^	5.05 ± 0.41 ^b^	6.5 ± 0.40 ^c^	8.06 ± 0.78 ^d^	4.81 ± 0.24 ^b^	8.15 ± 0.33 ^d^	8.89 ± 0.68 ^d^
Dietary Fibre (%)	15.2	0.00 ± 0.00	0.36 ± 0.09 ^a^	0.72 ± 0.10 ^b^	1.05 ± 0.05 ^c^	0.31 ± 0.06 ^a^	0.68 ± 0.10 ^b^	0.96 ± 0.05 ^c^

Data are presented as mean ± SD (*n* ≥ 3). Different alphabet superscripts indicate statistical difference (*p* < 0.05) across each row. POS5, POS10, and POS15: 5, 10, and 15% tamarillo powder was added post-fermentation, respectively. PRE5, PRE10, and PRE15: 5, 10, and 15% tamarillo powder was added to milk and starter culture prior to fermentation, respectively. TSS: total soluble solids.

**Table 2:** Rheological and textural parameters of yoghurt samples.

Parameters	Control	POS5	POS10	POS15	PRE5	PRE10	PRE15
Rheology							
Consistency coefficient (K, Pa·s)	<0.005	0.30 ± 0.03	2.22 ± 0.07	13.1 ± 0.37	0.28 ± 0.02	2.25 ± 0.07	10.54 ± 0.82
Flow behaviour index (n)	0.88 ± 0.12	0.68 ± 0.02	0.57 ± 0.01	0.40 ± 0.00	0.65 ± 0.01	0.52 ± 0.00	0.42 ± 0.01
Viscosity at 350 s^−1^ (Pa·s)	0.01 ± 0.00 ^a^	0.05 ± 0.00 ^b^	0.18 ± 0.01 ^c^	0.40 ± 0.01 ^d^	0.04 ± 0.00 ^b^	0.14 ± 0.01 ^e^	0.35 ± 0.01 ^f^
Elastic modulus (Pa)	N/A	0.16 ± 0.00 ^a^	0.57 ± 0.01 ^b^	1.05 ± 0.03 ^c^	0.12 ± 0.00 ^d^	0.45 ± 0.005 ^e^	0.93 ± 0.02 ^f^
Texture							
Firmness (N)	1.05 ± 0.00 ^a^	1.15 ± 0.01 ^b^	1.60 ± 0.02 ^c^	1.83 ± 0.08 ^d^	1.13 ± 0.02 ^b^	1.55 ± 0.02 ^c^	2.21 ± 0.04 ^e^
Consistency (N·s)	15.80 ± 0.04 ^a^	16.61 ± 0.05 ^a^	21.92 ± 0.46 ^b^	24.19 ± 0.87 ^c^	16.19 ± 0.10 ^a^	21.19 ± 0.37 ^b^	30.83 ± 1.07 ^d^
Cohesiveness (N)	<−0.005 ^a^	−0.14 ± 0.01 ^b^	−0.34 ± 0.00 ^c^	−0.5 ± 0.02 ^d^	−0.14 ± 0.01 ^b^	−0.28 ± 0.02 ^e^	−0.73 ± 0.05 ^f^

N/A: not applicable. Data are presented as mean ± SD (*n* ≥ 3). Different alphabet superscripts indicate statistical difference (*p* < 0.05) across each row. POS5, POS10, and POS15: 5, 10, and 15% of tamarillo powder was added post-fermentation, respectively. PRE5, PRE10, and PRE15: 5, 10, and 15% tamarillo powder was added to milk and starter culture prior to fermentation, respectively.

**Table 3:** Reducing sugar and organic acid profiles of yoghurt samples.

Analytes	Concentration (mg/100 g Yoghurt)						
	Control	POS5	POS10	POS15	PRE5	PRE10	PRE15
Reducing sugars							
Fructose	22.5 ± 3.08 ^a^	482 ± 120 ^b^	1066 ± 318 ^cd^	1161 ± 269 ^d^	365 ± 49.1 ^b^	909 ± 346 ^cd^	895 ± 156 ^c^
Lactose	1019 ± 86.4 ^a^	1483 ± 45.6 ^b^	1376 ± 91.4 ^c^	1263 ± 50.4 ^d^	2277 ± 31.2 ^e^	1938 ± 121 ^f^	1702 ± 25.4 ^g^
Galactose	283 ± 41.8 ^a^	387 ± 16.5 ^b^	372 ± 31.9 ^b^	318 ± 9.93 ^c^	46.8 ± 3.6 ^d^	44.5 ± 4.98 ^d^	36.4 ± 3.72 ^d^
Glucose	79.2 ± 11.5 ^a^	248 ± 11.1 ^b^	427 ± 30.8 ^c^	491 ± 26.9 ^d^	214 ± 9.99 ^e^	385 ± 30.3 ^f^	495 ± 22.7 ^d^
Mannose	1.72 ± 0.05 ^a^	3.49 ± 0.15 ^b^	4.21 ± 0.38 ^c^	4.54 ± 0.28 ^c^	4.66 ± 0.18 ^c^	5.79 ± 0.53 ^d^	6.12 ± 0.76 ^d^
Ribose	0.17 ± 0.01 ^a^	0.52 ± 0.08 ^b^	0.57 ± 0.06 ^b^	0.81 ± 0.13 ^c^	1.94 ± 0.3 ^d^	1.8 ± 0.4 ^de^	1.64 ± 0.27 ^e^
Rhamnose	n.d	<0.005 ^ab^	0.01 ± 0.01 ^ab^	0.05 ± 0.03 ^cd^	0.02 ± 0.02 ^abc^	0.03 ± 0.04 ^bc^	0.07 ± 0.04 ^d^
Maltose	739 ± 55.4 ^a^	1117 ± 131 ^b^	1070 ± 69 ^b^	1051 ± 73.3 ^b^	1643 ± 161 ^c^	1428 ± 163 ^d^	1340 ± 105 ^d^
Glucuronic acid	0.75 ± 0.05 ^a^	1.2 ± 0.08 ^b^	1.25 ± 0.16 ^b^	1.23 ± 0.17 ^b^	1.46 ± 0.2 ^c^	1.31 ± 0.27 ^bc^	1.31 ± 0.23 ^bc^
Arabinose	0.07 ± 0.03 ^a^	0.27 ± 0.04 ^b^	0.33 ± 0.06 ^b^	0.61 ± 0.09 ^c^	0.42 ± 0.05 ^d^	0.47 ± 0.1 ^de^	0.51 ± 0.1 ^e^
Xylose	0.04 ± 0.04 ^a^	1.95 ± 0.29 ^b^	3.46 ± 0.54 ^c^	3.91 ± 0.59 ^cd^	1.83 ± 0.31 ^b^	2.73 ± 0.32 ^e^	3.98 ± 0.53 ^d^
Fucose	0.24 ± 0.03 ^a^	0.31 ± 0.05 ^b^	0.31 ± 0.07 ^ab^	0.39 ± 0.06 ^c^	0.34 ± 0.05 ^bc^	0.34 ± 0.06 ^bc^	0.32 ± 0.05 ^b^
Total reducing sugars	2145 ± 198	3725 ± 325	4321 ± 543	4296 ± 431	4558 ± 256	4717 ± 668	4483 ± 315
Organic acids							
Malic acid	0.57 ± 0.09 ^a^	20.7 ± 0.57 ^b^	22.5 ± 0.18 ^c^	43.4 ± 0.13 ^d^	19.6 ± 0.33 ^b^	34.1 ± 0.72 ^e^	44.7 ± 1.09 ^f^
Lactic acid	674 ± 26.5 ^a^	920 ± 20.3 ^b^	1479 ± 124 ^c^	1683 ± 62.1 ^d^	846 ± 5.91 ^b^	1428 ± 20.6 ^c^	1833 ± 37.5 ^e^
Malonic acid	n.d	n.d	n.d	n.d	n.d	n.d	n.d
Citric acid	116 ± 13.2 ^a^	838 ± 12.5 ^b^	887 ± 27.6 ^c^	931 ± 62.9 ^c^	751 ± 16.9 ^d^	911 ± 32.6 ^c^	885 ± 23.5 ^c^
Succinic acid	n.d	n.d	n.d	n.d	n.d	n.d	n.d
Fumaric acid	n.d	n.d	n.d	n.d	n.d	n.d	0.72 ± 0.05 ^a^
Itaconic acid	n.d	0.28 ± 0.09 ^a^	0.81 ± 0.1 ^b^	0.91 ± 0.13 ^bc^	0.31 ± 0.03 ^a^	0.83 ± 0.11 ^b^	1.14 ± 0.1 ^c^
Vanillic acid	n.d	n.d	n.d	n.d	n.d	n.d	n.d
Syringic acid	n.d	1.9 ± 0.02 ^a^	2.28 ± 0.02 ^b^	2.74 ± 0.07 ^c^	1.2 ± 1.04 ^d^	2.27 ± 0.02 ^b^	2.63 ± 0.04 ^c^
Cinnamic acid	0.43 ± 0.01 ^a^	0.6 ± 0.05 ^b^	0.76 ± 0.06 ^c^	0.9 ± 0.06 ^d^	0.66 ± 0.08 ^b^	0.71 ± 0.02 ^c^	0.84 ± 0.05 ^d^
Total organic acid	791 ± 39.8	1782 ± 33.5	2392 ± 152	2662 ± 125	1619 ± 24.3	2377 ± 54.1	2768 ± 62.4

n.d: not detected. Data are presented as mean ± SD (*n* ≥ 3). Different alphabet superscripts indicate statistical difference (*p* < 0.05) across each row. POS5, POS10, and POS15: 5, 10, and 15% of tamarillo powder was added post-fermentation, respectively. PRE5, PRE10, and PRE15: 5, 10, and 15% tamarillo powder was added to milk and starter culture prior to fermentation, respectively.

**Table 4:** Concentrations and % RDI/AI of α-tocopherol, β-carotene, and ascorbic acid in yoghurt samples.

Yoghurt Samples	α-Tocopherol (mg/100 g Yoghurt)	%AI of α-Tocopherol	β-Carotene (mg/100 g Yoghurt)	%RDI of β-Carotene	Ascorbic Acid (mg/100 g Yoghurt)	%RDI of Ascorbic Acid
Control	0.09 ± 0 ^a^	1%	0.03 ± 0 ^a^	1%	0.57 ± 0.01 ^a^	2%
POS5	0.46 ± 0.02 ^b^	7%	0.17 ± 0.03 ^b^	5%	7.77 ± 0.33 ^b^	**26%**
POS10	0.62 ± 0.01 ^c^	9%	0.26 ± 0.01 ^c^	7%	11.57 ± 0.25 ^c^	**39%**
POS15	0.89 ± 0.02 ^d^	**13%**	0.35 ± 0.01 ^d^	**10%**	15.85 ± 0.35 ^d^	**53%**
PRE5	0.41 ± 0.01 ^e^	6%	0.15 ± 0.02 ^b^	4%	2.49 ± 0.02 ^e^	8%
PRE10	0.72 ± 0.01 ^f^	**11%**	0.24 ± 0.01 ^e^	7%	6.27 ± 0.17 ^f^	21%
PRE15	0.97 ± 0.04 ^g^	**15%**	0.34 ± 0.03 ^d^	9%	7.77 ± 0.05 ^b^	**26%**

Data are presented as mean ± SD (*n* ≥ 3). Different alphabet superscripts indicate statistical difference (*p* < 0.05) across each column. RDI: recommended dietary intake. AI: adequate intake (used when an RDI cannot be determined). 1 µg retinol (vitamin A) equivalent = 6 µg all trans β carotene RDI = 900 retinol equivalents. Vitamin E: AI = 10 mg, UL = 300 mg. Vitamin C: estimated average requirement (EAR) = 30 mg, RDI = 45 mg.–: not identified. **Bolded** numbers are a dietary source (>10% RDI) and **bolded and underlined ** numbers are a good source (>25% RDI) [2]. The RDI and AI were calculated based on mg/serving, in which serving size was 150 g.

## Supplementary Materials

The following supporting information can be downloaded at: https://www.mdpi.com/article/10.3390/foods11010079/s1, Table S1: Volatile compounds and their relative contents in control and tamarillo yoghurts, Table S2: Relative content percentage (%) of volatile classes identified in yoghurt samples, Table S3: Fatty acid profiles and lipid indices of control and tamarillo fortified yoghurts.
