# Supplementary material for: Effect of Tamarillo Fortification and Fermentation Process on Physicochemical Properties and Nutrient and Volatiles Content of Yoghurt
Source: Foods. 2021 Dec 29;11(1):79. doi: 10.3390/foods11010079 (PMC8750935; doi:10.3390/foods11010079)
Supplement: Supplementary file 1 [file foods-11-00079-s001.zip › foods-1498539-supplementary.pdf]

Table S1. Volatile compounds and their relative contents in control and tamarillo yoghurts.

No.	Compounds	RI	Relative Concentration (µg/g yoghurt)						
			Control	POS5	POS10	POS15	PRE5	PRE10	PRE15
Acids									
1	Propanoic acid, anhydride	1162.0	0.01 ± 0a	0.19 ± 0.01bc	0.2 ± 0.02b	0.16 ± 0.01c	0.24 ± 0.01d	0.37 ± 0.04e	0.36 ± 0.01e
2	Acetic acid	1448.5	3.81 ± 0.2a	2.34 ± 0.07b	2.14 ± 0.13bc	2.06 ± 0.23c	0.88 ± 0.05d	0.88 ± 0.11d	0.83 ± 0.03d
3	Propanoic acid	1538.9	0.06 ± 0a	0.03 ± 0b	0.03 ± 0b	0.03 ± 0b	0.02 ± 0c	0.02 ± 0d	0.02 ± 0cd
4	Butanoic acid	1627.3	13.33 ± 0.64a	5.75 ± 0.17b	5.38 ± 0.06b	5.22 ± 0.05b	3.29 ± 0.14c	3.37 ± 0.13cd	3.91 ± 0.47d
5	3-Methyl-Butanoic acid	1671.7	0.16 ± 0ab	0.18 ± 0.01c	0.17 ± 0bc	0.17 ± 0.01bc	0.11 ± 0.01e	0.15 ± 0.02ad	0.15 ± 0d
6	Pentanoic acid	1740.0	0.2 ± 0.01a	0.07 ± 0b	0.07 ± 0b	0.07 ± 0b	0.05 ± 0c	0.06 ± 0.01c	0.06 ± 0c
7	3-Methyl-2-Butenoic acid	1797.5	0.01 ± 0a	0.01 ± 0b	0.02 ± 0c	0.02 ± 0d	0.01 ± 0a	0.01 ± 0e	0.01 ± 0f
8	3-Methyl-3-Butenoic acid	1797.5	n.d	0.01 ± 0a	0.06 ± 0b	0.03 ± 0c	< 0.005d	0.01 ± 0e	0.02 ± 0f
9	Hexanoic acid	1845.9	18.33 ± 0.77a	8.17 ± 0.25b	7.39 ± 0.08bc	7.21 ± 0.27c	4.71 ± 0.26d	5.12 ± 0.19d	5.33 ± 0.89d
10	Heptanoic acid	1956.0	0.22 ± 0a	0.07 ± 0b	0.06 ± 0bc	0.06 ± 0.01bc	0.06 ± 0.01bc	0.05 ± 0.02c	0.05 ± 0c
11	(E)-2-Hexenoic acid	1972.6	< 0.005a	0.06 ± 0b	0.06 ± 0.01bc	0.03 ± 0d	0.05 ± 0.01b	0.05 ± 0.01b	0.07 ± 0c
12	Octanoic acid	2098.2	4.92 ± 0.35a	1.62 ± 0.09b	1.24 ± 0.05c	1.24 ± 0.09c	0.66 ± 0.11d	0.7 ± 0.2d	0.82 ± 0.02d
13	Nonanoic acid	2386.1	0.08 ± 0.03a	0.05 ± 0b	0.03 ± 0.01bc	0.01 ± 0c	0.04 ± 0.01b	0.03 ± 0bc	0.01 ± 0c
14	n-Decanoic acid	2658.3	0.52 ± 0.06a	0.14 ± 0.01b	0.1 ± 0.01c	0.12 ± 0.01bc	0.05 ± 0.01d	0.05 ± 0.02d	0.08 ± 0.01e
15	Benzoic acid	3105.6	3.07 ± 0.59a	2.01 ± 0.31b	1.76 ± 0.15b	1.58 ± 0.11b	0.14 ± 0.04c	0.12 ± 0.03c	0.07 ± 0.01c
Alcohols									
16	Ethanol	913.5	0.01 ± 0.01a	0.82 ± 0.03b	1.72 ± 0.16c	0.7 ± 0.06b	0.44 ± 0.09d	1.01 ± 0.1e	1.07 ± 0.05e
17	1-Butanol	1149.3	0.06 ± 0a	0.06 ± 0ab	0.05 ± 0b	0.05 ± 0.01b	0.04 ± 0c	0.04 ± 0.01c	0.04 ± 0c
18	3-Methyl-1-Butanol	1210.7	0.1 ± 0.01a	0.11 ± 0.01a	0.12 ± 0.01a	0.07 ± 0.01a	0.28 ± 0.1b	0.1 ± 0.02a	0.25 ± 0.05b
19	3-Methyl-3-Buten-1-ol	1251.3	0.09 ± 0a	0.33 ± 0.01b	0.44 ± 0.03c	0.22 ± 0.02d	0.18 ± 0.04d	0.38 ± 0.05e	0.49 ± 0.01f
20	1-Pentanol	1253.4	1.23 ± 0.1a	0.08 ± 0b	0.07 ± 0b	0.07 ± 0b	0.1 ± 0.01b	0.05 ± 0.01b	0.08 ± 0b

21	Prenol	1323.8	0.22 ± 0.06a	0.14 ± 0b	0.23 ± 0.01a	0.13 ± 0.01b	0.06 ± 0.01c	0.1 ± 0.01bc	0.12 ± 0.01b
22	3-Pentanol	1344.1	5.03 ± 0.27a	2.24 ± 0.06b	2.14 ± 0.06b	2.48 ± 0.09c	0.01 ± 0d	< 0.005d	< 0.005d
23	1-Hexanol	1356.0	0.2 ± 0.01ab	0.19 ± 0.01a	0.35 ± 0.01c	0.28 ± 0.02bc	1.67 ± 0.1d	0.59 ± 0.06e	0.9 ± 0.03f
24	(E)-3-Hexen-1-ol	1366.1	< 0.005a	0.01 ± 0b	0.02 ± 0c	0.02 ± 0d	0.04 ± 0e	0.02 ± 0f	0.02 ± 0df
25	(Z)-3-Hexen-1-ol	1386.7	0.01 ± 0a	0.27 ± 0.01b	0.84 ± 0.03c	0.42 ± 0.03d	0.21 ± 0.01e	0.37 ± 0.04f	0.44 ± 0.01d
26	(Z)-2-Hexen-1-ol	1408.0	< 0.005a	0.01 ± 0a	0.01 ± 0a	0.01 ± 0a	0.1 ± 0.01b	0.03 ± 0c	0.09 ± 0.02b
27	1-Heptanol	1458.2	0.16 ± 0.01a	0.06 ± 0b	0.06 ± 0b	0.06 ± 0.01b	0.02 ± 0c	0.01 ± 0d	0.02 ± 0cd
28	2,6-Dimethyl-4-Heptanol	1474.2	0.04 ± 0a	0.02 ± 0b	0.02 ± 0b	0.02 ± 0c	< 0.005d	n.d	n.d
29	2,3-Butanediol	1542.0	0.12 ± 0.02a	0.09 ± 0b	0.09 ± 0.01b	0.27 ± 0c	0.06 ± 0.01d	0.09 ± 0.02b	0.13 ± 0.01a
30	1-Octanol	1560.6	0.03 ± 0a	0.01 ± 0b	0.01 ± 0b	0.01 ± 0b	0.01 ± 0c	0.01 ± 0c	0.01 ± 0c
31	Terpinen-4-ol	1606.7	n.d	0.01 ± 0a	0.02 ± 0b	0.01 ± 0a	0.01 ± 0a	0.01 ± 0a	0.02 ± 0b
32	1-Nonanol	1662.9	0.04 ± 0a	0.01 ± 0b	0.01 ± 0b	0.01 ± 0b	0.01 ± 0c	0.01 ± 0c	0.01 ± 0c
33	Alpha-Terpineol	1701.3	< 0.005a	0.04 ± 0b	0.05 ± 0c	0.07 ± 0d	0.04 ± 0b	0.08 ± 0.01e	0.1 ± 0f
34	p-Mentha-1,5-dien-8-ol	1729.5	n.d	0.04 ± 0a	0.07 ± 0b	0.18 ± 0.02c	0.02 ± 0d	0.09 ± 0.01b	0.16 ± 0e
35	p-Mentha-1(7),2-dien-8-ol	1785.4	n.d	0.06 ± 0a	0.11 ± 0b	0.29 ± 0.03c	0.04 ± 0d	0.12 ± 0.02b	0.22 ± 0.01e
36	Phenylethyl Alcohol	1914.8	0.02 ± 0a	0.02 ± 0a	0.01 ± 0a	0.01 ± 0a	0.06 ± 0.01b	0.02 ± 0a	0.05 ± 0.02b
37	[R-(R*,R*)]-1,2-diphenyl-1,2-Ethanediol	2169.4	< 0.005a	0.01 ± 0b	0.01 ± 0b	0.01 ± 0d	0.01 ± 0b	0.01 ± 0c	0.01 ± 0c
Aldehydes									
38	Acetaldehyde	629.8	1.04 ± 0.09a	0.12 ± 0.04a	0.55 ± 0.05b	0.79 ± 0.11c	0.22 ± 0.02d	0.16 ± 0.01d	0.13 ± 0.01d
39	Butanal	823.6	0.03 ± 0a	0.01 ± 0b	0.01 ± 0b	0.01 ± 0c	0.01 ± 0bc	0.01 ± 0d	0.01 ± 0d
40	2-Methyl-Butanal	876.3	0.04 ± 0a	0.11 ± 0.02a	0.22 ± 0.02b	0.29 ± 0.04b	0.24 ± 0.03b	0.89 ± 0.13c	0.99 ± 0.07c
41	3-Methyl- Butanal	883.1	0.04 ± 0a	0.14 ± 0ab	0.35 ± 0.04bc	0.47 ± 0.03c	0.53 ± 0.07c	2.29 ± 0.33d	2.39 ± 0.05d
42	Hexanal	1069.9	0.09 ± 0.01a	0.17 ± 0.01b	0.11 ± 0.01a	0.05 ± 0.01c	0.11 ± 0a	0.56 ± 0.05d	0.62 ± 0.02e
43	3-Methyl-2-Butenal	1195.3	0.24 ± 0.02a	0.27 ± 0.01b	0.33 ± 0.02c	0.48 ± 0.02d	0.09 ± 0.01e	0.16 ± 0.02f	0.15 ± 0f
44	(E)-2-Hexenal	1214.4	0.01 ± 0a	1.08 ± 0.09b	0.78 ± 0.03c	0.3 ± 0.05d	0.54 ± 0.05e	1.3 ± 0.21f	1.33 ± 0.09f

45	Nonanal	1392.5	0.03 ± 0.01a	0.01 ± 0b	0.01 ± 0bc	0.01 ± 0bc	0.01 ± 0b	0.01 ± 0b	0.02 ± 0c
46	(E,E)-2,4-Hexadienal	1396.1	0.01 ± 0a	0.04 ± 0b	0.03 ± 0c	0.01 ± 0d	0.02 ± 0e	0.04 ± 0b	0.05 ± 0f
Benzenes									
47	Toluene	1025.3	0.44 ± 0.09ab	0.41 ± 0.05a	0.66 ± 0.01bc	1.3 ± 0.18d	0.27 ± 0.07a	0.7 ± 0.25c	1.3 ± 0.09d
48	1,3-Dimethyl-Benzene	1126.4	0.03 ± 0.01a	0.02 ± 0b	0.03 ± 0a	0.03 ± 0.01a	< 0.005c	0.01 ± 0b	0.03 ± 0a
49	1-Methyl-3-(1-methylethyl)-Benzene	1265.2	0.01 ± 0a	0.04 ± 0bc	0.06 ± 0.01c	0.11 ± 0.03d	0.03 ± 0.01ab	0.05 ± 0.01c	0.1 ± 0.01d
50	1,2,3-Trimethyl-Benzene	1276.1	0.02 ± 0a	0.01 ± 0b	0.02 ± 0a	0.02 ± 0a	0.01 ± 0c	0.01 ± 0b	0.02 ± 0a
51	1-Methyl-3-(1-methylethenyl)-Benzene	1436.1	0.01 ± 0a	0.01 ± 0ab	0.02 ± 0b	0.04 ± 0.01c	0.01 ± 0a	0.02 ± 0.01b	0.03 ± 0c
52	1-Methyl-4-(1-methylethenyl)-Benzene	1436.1	0.01 ± 0ab	0.02 ± 0c	0.02 ± 0c	0.03 ± 0.01d	0.01 ± 0a	0.02 ± 0bc	0.03 ± 0d
53	Benzaldehyde	1523.2	0.08 ± 0ab	0.08 ± 0ab	0.1 ± 0.01bc	0.12 ± 0c	0.07 ± 0.01a	0.16 ± 0.03d	0.22 ± 0.01e
54	Benzonitrile	1606.2	0.03 ± 0a	0.01 ± 0b	0.01 ± 0b	0.01 ± 0b	0.01 ± 0c	0.01 ± 0c	0.01 ± 0c
55	Benzeneacetaldehyde	1641.3	0.03 ± 0.01a	0.15 ± 0.02b	0.29 ± 0.02c	0.27 ± 0c	0.29 ± 0.02c	1.3 ± 0.19d	1.53 ± 0.05e
56	3-Ethyl-Benzaldehyde	1710.3	0.01 ± 0a	< 0.005b	< 0.005b	< 0.005b	< 0.005b	< 0.005b	< 0.005b
57	Methoxy-phenyl-Oxime	1756.6	3.05 ± 0.56a	1.08 ± 0.14b	0.96 ± 0.1b	0.79 ± 0.13b	0.91 ± 0.34b	0.79 ± 0.18b	0.68 ± 0.12b
58	Meso-Hydrobenzoin	1878.3	< 0.005ab	0.01 ± 0a	0.01 ± 0c	0.01 ± 0d	< 0.005b	0.01 ± 0e	0.01 ± 0d
59	2-Methyl-Phenol	2001.9	< 0.005a	0.03 ± 0b	0.04 ± 0c	0.06 ± 0d	0.01 ± 0e	0.03 ± 0f	0.04 ± 0c
Esters									
60	Butanoic acid, methyl ester	965.7	0.01 ± 0a	1.42 ± 0.05b	2.25 ± 0.43c	4.74 ± 0.56d	0.95 ± 0.03b	2.19 ± 0.22c	2.13 ± 0.07c
61	Butanoic acid, ethyl ester	1024.9	n.d	0.13 ± 0a	0.49 ± 0.07b	0.77 ± 0.02c	0.51 ± 0.02b	1.1 ± 0.11d	1.35 ± 0.06e
62	Isopropyl butyrate	1029.9	0.01 ± 0a	0.23 ± 0.01b	0.18 ± 0.03c	0.05 ± 0d	0.14 ± 0.01e	0.33 ± 0.04f	0.43 ± 0.01g
63	3-Butenoic acid, 3-methyl-, methyl ester	1110.8	n.d	0.03 ± 0a	0.05 ± 0.01b	0.2 ± 0.01c	0.01 ± 0.01d	0.04 ± 0be	0.05 ± 0b
64	2-Butenoic acid, 3-methyl-, methyl ester	1161.3	n.d	0.12 ± 0.01ab	0.41 ± 0.06c	1.3 ± 0.06d	0.08 ± 0a	0.18 ± 0.02be	0.22 ± 0.01e
65	Hexanoic acid, methyl ester	1181.7	0.17 ± 0.08a	8.17 ± 0.42b	11.22 ± 2.5c	12.74 ± 0.82cd	6.19 ± 0.44b	10.84 ± 1.68c	13.69 ± 1.01d
66	3-Methyl-3-buten-1-ol, acetate	1190.8	0.05 ± 0.01a	1.69 ± 0.04b	1 ± 0.04c	0.62 ± 0.07d	0.62 ± 0.08d	1.3 ± 0.15e	1.65 ± 0.03b
67	Hexanoic acid, ethyl ester	1230.7	n.d	0.07 ± 0.01a	0.19 ± 0.05b	0.2 ± 0.02b	0.22 ± 0.01b	0.4 ± 0.06c	0.74 ± 0.05d

68	4-Hexenoic acid, methyl ester	1256.6	n.d	0.01 ± 0a	0.01 ± 0b	0.01 ± 0c	0.01 ± 0c	0.01 ± 0d	0.02 ± 0e
69	Acetic acid, methyl ester	1299.5	0.07 ± 0.01a	0.03 ± 0b	0.03 ± 0cd	0.03 ± 0bc	0.03 ± 0de	0.02 ± 0e	0.02 ± 0f
70	Butanoic acid, 4-pentenyl ester	1339.8	0.01 ± 0a	0.18 ± 0.01b	0.26 ± 0.04c	0.58 ± 0.02d	0.15 ± 0.01b	0.29 ± 0.05c	0.52 ± 0.03e
71	Octanoic acid, methyl ester	1389.5	n.d	0.02 ± 0a	0.03 ± 0b	0.04 ± 0c	0.02 ± 0a	0.03 ± 0.01b	0.06 ± 0d
72	Ethylene glycol di-n-butyrate	1515.1	n.d	0.02 ± 0a	0.08 ± 0b	0.03 ± 0c	0.01 ± 0d	0.03 ± 0c	0.04 ± 0e
73	Butanedioic acid, diethyl ester	1567.6	n.d	0.01 ± 0a	0.03 ± 0b	0.01 ± 0a	0.01 ± 0c	0.02 ± 0d	0.02 ± 0b
74	Hexanoic acid, 2-hydroxy-, methyl ester	1580.4	n.d	0.03 ± 0a	0.05 ± 0b	0.08 ± 0c	0.03 ± 0a	0.06 ± 0.01d	0.09 ± 0e
75	Benzoic acid, methyl ester	1624.2	0.03 ± 0.01a	0.1 ± 0.01b	0.14 ± 0.01c	0.24 ± 0.01d	0.07 ± 0.01e	0.15 ± 0.02c	0.22 ± 0f
76	Hexanoic acid, 4-oxo-, methyl ester	1643.3	n.d	0.07 ± 0a	0.12 ± 0b	0.09 ± 0c	0.04 ± 0d	0.11 ± 0.02b	0.17 ± 0.01e
77	Propanoic acid, 2-methyl-, ethyl ester	1692.8	n.d	0.04 ± 0a	0.13 ± 0b	0.12 ± 0c	0.02 ± 0d	0.06 ± 0.01e	0.09 ± 0f
78	Methyl salicylate	1778.8	0.02 ± 0.01a	0.05 ± 0b	0.07 ± 0c	0.07 ± 0.01c	0.03 ± 0a	0.05 ± 0.01b	0.06 ± 0c
Furans									
79	2-Pentyl-Furan	1225.0	0.01 ± 0a	0.02 ± 0ab	0.02 ± 0ab	0.1 ± 0.02c	0.07 ± 0d	0.02 ± 0.01ab	0.03 ± 0.01b
80	Furfural	1461.6	0.02 ± 0a	0.01 ± 0b	0.01 ± 0b	0.02 ± 0b	0.01 ± 0b	0.03 ± 0b	0.03 ± 0b
81	2-Acetyl-5-methylfuran	1663.2	n.d	0.01 ± 0a	0.01 ± 0b	0.01 ± 0b	< 0.005c	0.01 ± 0a	0.01 ± 0b
82	2-Vinylfuran	2006.0	0.03 ± 0a	0.02 ± 0b	0.01 ± 0c	0.01 ± 0c	0.01 ± 0d	0.01 ± 0c	0.01 ± 0c
Hydrocarbons									
83	3-Methylenecyclohexene	927.1	n.d	0.05 ± 0ab	0.11 ± 0c	0.22 ± 0.04d	0.03 ± 0a	0.07 ± 0.01b	0.12 ± 0c
84	4-methyl-1-(1-methylethyl)- Bicyclo [3.1.0] hex-2-ene	1088.5	<0.005a	0.01 ± 0a	0.01 ± 0.01a	0.01 ± 0.01a	n.d	0.01 ± 0.01a	0.01 ± 0.01a
85	2,2-Dimethylpropanoic anhydride	1376.5	n.d	0.02 ± 0a	0.02 ± 0b	< 0.005c	0.01 ± 0d	0.03 ± 0e	0.04 ± 0f
86	5-Ethyldecane	1600.1	0.05 ± 0a	0.02 ± 0b	0.02 ± 0b	0.02 ± 0b	0.01 ± 0c	0.01 ± 0c	0.01 ± 0c
87	2,6-Dimethyl-2-trans-6-octadiene	2154.3	n.d	0.02 ± 0a	0.02 ± 0b	0.03 ± 0c	0.01 ± 0d	0.02 ± 0b	0.03 ± 0c
Ketones									
88	Acetone	726.0	5.93 ± 0.29a	3.28 ± 0.08b	3.22 ± 0.27b	2.23 ± 0.21c	2.4 ± 0.23cd	2.67 ± 0.22d	1.69 ± 0.05e

89	2-Butanone	857.6	3.7 ± 0.23a	1.21 ± 0.06b	1.17 ± 0.08b	0.77 ± 0.08c	0.88 ± 0.05c	0.9 ± 0.12c	0.52 ± 0.05d
90	2,3-Butanedione	953.9	7.97 ± 0.58a	3.71 ± 0.22b	3.25 ± 0.19c	3.56 ± 0.12bc	0.66 ± 0.05d	0.08 ± 0.01e	0.06 ± 0.01e
91	2,3-Pentanedione	1046.7	1.34 ± 0.11a	0.57 ± 0.01b	0.48 ± 0.03c	0.93 ± 0.06d	0.09 ± 0.01e	0.01 ± 0e	0.01 ± 0e
92	2-Heptanone	1177.4	0.08 ± 0.01a	0.03 ± 0b	0.04 ± 0c	0.03 ± 0.01bc	0.01 ± 0d	0.02 ± 0d	0.01 ± 0d
93	Acetoin	1286.8	28.62 ± 1.43a	14.12 ± 0.32b	12.7 ± 0.42c	10.84 ± 0.3d	0.9 ± 0.04e	0.03 ± 0e	0.03 ± 0e
94	6-Methyl-5-Hepten-2-one	1337.0	0.01 ± 0a	0.01 ± 0b	0.02 ± 0c	0.01 ± 0d	0.01 ± 0b	0.02 ± 0e	0.03 ± 0f
95	2-Hydroxy-3-pentanone	1360.1	8.49 ± 0.43a	3.83 ± 0.11bc	3.67 ± 0.11b	4.22 ± 0.12c	5.65 ± 0.34d	2.01 ± 0.21e	3.04 ± 0.11f
96	3-(hydroxymethyl)-2-Nonanone	1389.1	0.19 ± 0.04a	0.06 ± 0b	0.06 ± 0b	0.07 ± 0.01b	0.03 ± 0c	0.03 ± 0c	0.03 ± 0c
Nitrogen compounds									
97	2-nitro-Propane	1117.3	0.06 ± 0.01a	0.06 ± 0ab	0.05 ± 0.01ab	0.03 ± 0.01cd	0.03 ± 0c	0.04 ± 0.02bd	0.03 ± 0c
98	Bromochloronitromethane	1293.4	0.01 ± 0a	< 0.005b	< 0.005b	< 0.005b	< 0.005b	< 0.005b	< 0.005b
99	4-Cyanocyclohexene	1566.5	0.01 ± 0a	0.01 ± 0a	0.02 ± 0b	0.02 ± 0b	0.01 ± 0c	0.01 ± 0d	0.02 ± 0b
Pyrans									
100	Tetrahydro-2H-Pyran-2-methanol	990.6	n.d	0.05 ± 0a	0.1 ± 0b	0.04 ± 0.01a	0.03 ± 0c	0.07 ± 0.01d	0.09 ± 0e
101	Tetrahydro-6-pentyl- 2H-Pyran-2-one	2470.0	0.24 ± 0.02a	0.08 ± 0b	0.07 ± 0bc	0.06 ± 0bc	0.04 ± 0.01d	0.06 ± 0.01cd	0.05 ± 0cd
Sulphur compounds									
102	Ethanethiol	659.4	0.07 ± 0a	0.14 ± 0.01b	0.24 ± 0.02c	0.23 ± 0.01c	0.17 ± 0.01d	0.42 ± 0.03e	0.39 ± 0.01f
103	Dihydro-2-methyl-3(2H)-Thiophenone	1530.2	0.4 ± 0.03a	0.09 ± 0b	0.07 ± 0c	0.06 ± 0c	n.d	n.d	n.d
104	Dimethyl sulfone	1899.3	0.35 ± 0.08a	0.21 ± 0.03b	0.12 ± 0.01c	0.13 ± 0.01c	0.14 ± 0.03c	0.08 ± 0.02c	0.09 ± 0.01c
Terpenes									
105	Limonene	1186.6	0.01 ± 0a	0.03 ± 0.01b	0.06 ± 0c	0.06 ± 0c	0.03 ± 0b	0.04 ± 0.01b	0.07 ± 0.01d
106	Eucalyptol	1204.2	n.d	0.1 ± 0a	0.2 ± 0b	0.09 ± 0.01ac	0.08 ± 0.01c	0.15 ± 0.02d	0.24 ± 0e
107	γ-Terpinene	1240.0	n.d	< 0.005a	< 0.005b	< 0.005bc	< 0.005d	< 0.005a	< 0.005c

* n.d.: not detected; RI: retention index; *m/z*: mass-to-charge ratio. Data are presented as Mean ± SD (n = 3) and listed in the order of group and then retention index as determined using the homologous series of n-alkanes and found in library. Different alphabets indicate statistical difference ($p < 0.05$) across each row.

Table S2. Relative content percentage (%) of volatile classes identified in yoghurt samples

Chemical class	Relative content percentage (%)						
	Control	POS5	POS10	POS15	PRE5	PRE10	PRE15
Acids	38.76	29.44	25.89	13.68	27.45	23.54	21.88
Alcohols	6.39	6.58	8.93	4.09	9.08	6.73	7.85
Aldehydes	1.33	4.20	3.31	1.83	4.71	11.61	10.56
Benzenes	3.23	2.66	3.07	2.12	4.31	6.66	7.42
Esters	0.32	17.66	23.14	60.23	24.33	36.78	39.87
Furans	0.05	0.09	0.07	0.11	0.24	0.15	0.15
Hydrocarbons	0.04	0.17	0.25	0.21	0.16	0.30	0.39
Ketones	48.88	38.11	34.06	17.18	28.30	12.36	10.06
Nitrogen and sulphur compounds	0.78	0.73	0.69	0.36	0.93	1.18	0.98
Terpenes and pyrans	0.22	0.37	0.60	0.19	0.48	0.69	0.84

Table S3. Fatty acid profiles and lipid indices of control and tamarillo fortified yoghurts.

Fatty acids	Formula	Concentration (mg/100 g yoghurt)						
		Control	POS5	POS10	POS15	PRE5	PRE10	PRE15
Saturated fatty acids (SFAs)								
Butyric acid	C4:0	35.98 ± 8.79^{ab}	40.07 ± 7.7^{a}	34.36 ± 3.45^{ab}	34.84 ± 10.08^{ab}	31.21 ± 2.69^{bc}	25.67 ± 2.27^{c}	23.81 ± 4.55^{c}
Hexanoic acid	C6:0	19.52 ± 4.35^{ac}	23.3 ± 1.41^{b}	22.02 ± 3.38^{ab}	22.5 ± 5.28^{ab}	18.82 ± 1.42^{ac}	16.09 ± 1.26^{cd}	14.61 ± 2.56^{d}
Octanoic acid	C8:0	11.87 ± 2.89^{ac}	15.17 ± 1.31^{b}	15.54 ± 2.59^{b}	13.99 ± 2.72^{ab}	12.38 ± 0.9^{ac}	10.2 ± 0.82^{cd}	9.49 ± 1.47^{d}
Decanoic acid	C10:0	25.33 ± 6.88^{a}	32.94 ± 2.28^{b}	34.09 ± 4.72^{b}	27.29 ± 4.86^{a}	26.53 ± 2.07^{a}	20.46 ± 1.07^{c}	19.35 ± 2.65^{c}
Undecanoic acid	C11:0	0.44 ± 0.12^{a}	0.56 ± 0.02^{b}	0.57 ± 0.07^{b}	0.44 ± 0.08^{a}	0.46 ± 0.04^{a}	0.34 ± 0.01^{c}	0.33 ± 0.04^{c}
Dodecanoic acid	C12:0	48.34 ± 12.74^{a}	64.97 ± 1.1^{b}	64.91 ± 6.57^{b}	48.06 ± 8.7^{a}	49.46 ± 4.18^{a}	36.55 ± 1.26^{c}	34.85 ± 4^{c}
Tridecanoic acid	C13:0	1.03 ± 0.18^{a}	1.2 ± 0.05^{b}	1.17 ± 0.07^{b}	0.95 ± 0.12^{a}	1.01 ± 0.07^{a}	0.83 ± 0.02^{c}	0.81 ± 0.05^{c}
Myristic acid	C14:0	98.12 ± 23.3^{a}	125.67 ± 11.27^{b}	119.26 ± 9.82^{b}	87.64 ± 16.87^{a}	96.04 ± 8.18^{a}	70.72 ± 3.83^{c}	66.85 ± 6.82^{c}
Pentadecanoic acid	C15:0	8.67 ± 1.92^{a}	10.75 ± 1.26^{b}	10.08 ± 0.78^{b}	7.35 ± 1.41^{ac}	8.35 ± 0.8^{a}	6.17 ± 0.43^{cd}	5.93 ± 0.58^{d}
Palmitic acid	C16:0	196.93 ± 40.37^{a}	248.27 ± 31.76^{b}	234.36 ± 17.21^{b}	172.12 ± 32.19^{ac}	184.79 ± 16.26^{a}	142.49 ± 11.18^{cd}	134.79 ± 16.31^{d}
Heptadecanoic acid	C17:0	4.28 ± 0.82^{ab}	5.25 ± 0.84^{c}	4.89 ± 0.36^{bc}	3.5 ± 0.61^{de}	3.96 ± 0.38^{ad}	3.06 ± 0.26^{e}	2.92 ± 0.35^{e}
Stearic acid	C18:0	81.77 ± 17.01^{a}	103.65 ± 17.18^{b}	96.08 ± 8.4^{b}	65.35 ± 12.58^{cd}	74.28 ± 8.01^{ac}	55.61 ± 5.59^{d}	52.9 ± 8.2^{d}
Arachidic acid	C20:0	0.85 ± 0.15^{ab}	1.18 ± 0.18^{c}	1.27 ± 0.13^{c}	0.9 ± 0.17^{a}	0.84 ± 0.09^{ab}	0.74 ± 0.05^{b}	0.79 ± 0.12^{ab}
Heneicosanoic acid	C21:0	0.2 ± 0.03^{a}	0.23 ± 0.04^{b}	0.24 ± 0.02^{b}	0.16 ± 0.03^{c}	0.16 ± 0.02^{c}	0.14 ± 0.02^{c}	0.13 ± 0.02^{c}
Behenic acid	C22:0	0.3 ± 0.06^{a}	0.57 ± 0.09^{b}	0.72 ± 0.09^{c}	0.56 ± 0.12^{b}	0.4 ± 0.04^{d}	0.4 ± 0.04^{d}	0.45 ± 0.09^{d}
Tricosanoic acid	C23:0	0.19 ± 0.03^{a}	0.26 ± 0.04^{bc}	0.29 ± 0.05^{b}	0.23 ± 0.03^{c}	0.19 ± 0.02^{a}	0.19 ± 0.03^{a}	0.19 ± 0.02^{a}
Lignoceric acid	C24:0	1.13 ± 0.06^{a}	1.48 ± 0.08^{bc}	1.75 ± 0.11^{d}	1.61 ± 0.16^{c}	1.3 ± 0.06^{e}	1.33 ± 0.16^{e}	1.43 ± 0.1^{be}
Monounsaturated fatty acids (MUFAs)								
Myristoleic acid	C14:1	7.43 ± 1.87^{a}	9.09 ± 0.48^{b}	9.02 ± 0.87^{b}	6.77 ± 1.23^{a}	7.33 ± 0.64^{a}	5.44 ± 0.22^{c}	5.16 ± 0.53^{c}
cis-10-Pentadecenoic acid	C15:1	n.d.	n.d.	n.d.	n.d.	n.d.	n.d.	n.d.
Palmitoleic acid	C16:1	13.33 ± 3.27^{a}	17.19 ± 2.01^{b}	17.03 ± 1.32^{b}	12.29 ± 2.48^{a}	12.87 ± 1.13^{a}	9.33 ± 0.71^{c}	9.1 ± 1.11^{c}

cis-10-Heptadecenoic acid	C17:1	n.d.	n.d.	n.d.	n.d.	n.d.	n.d.	n.d.
Elaidic, Oleic	C18:1 c+t	143.63 ± 31.91[ab]	199.55 ± 29.6[c]	202.5 ± 14.25[c]	156.91 ± 24.19[a]	157.16 ± 12.89[a]	122.84 ± 10.51[b]	121.52 ± 17.39[b]
cis-11-Eicosenoic acid	C20:1	n.d.	n.d.	n.d.	n.d.	n.d.	n.d.	n.d.
Erucic acid	C22:1	n.d.	n.d.	n.d.	n.d.	n.d.	n.d.	n.d.
cis-15-Tetracosenoic acid	C24:1	n.d.	n.d.	n.d.	n.d.	n.d.	n.d.	n.d.
Polyunsaturated fatty acids (PUFAs)								
Linoleaidic, Linoleic	C18:2 c+t	7.7 ± 1.72[a]	60.85 ± 9.65[bc]	105.72 ± 8.39[d]	86.02 ± 18.7[e]	38.25 ± 2.83[f]	53.72 ± 4.68[b]	65.5 ± 9.68[c]
γ-Linolenic acid	C18:3 n-6	n.d.	n.d.	n.d.	n.d.	n.d.	n.d.	n.d.
Linolenic acid	C18:3 n-3	4.44 ± 1.01[a]	9 ± 0.84[b]	11.74 ± 1.16[c]	9.6 ± 2.2[b]	6.27 ± 0.46[d]	6.34 ± 0.31[d]	6.63 ± 1.02[d]
cis-11,14-Eicosadienoic acid	C20:2	n.d.	n.d.	n.d.	n.d.	n.d.	n.d.	n.d.
cis-8,11,14-Eicosatrienoic acid	C20:3 n-6	0.25 ± 0.09[a]	0.34 ± 0.07[b]	0.32 ± 0.05[b]	0.19 ± 0.05[ac]	0.22 ± 0.03[a]	0.14 ± 0.03[cd]	0.11 ± 0.03[d]
Arachidonic acid	C20:4	0.63 ± 0.17[bc]	0.76 ± 0.08[a]	0.74 ± 0.1[ab]	0.47 ± 0.1[de]	0.52 ± 0.06[cd]	0.38 ± 0.03[ef]	0.34 ± 0.07[f]
11,14,17-Eicosatrienoic acid	C20:3 n-3	n.d.	n.d.	n.d.	n.d.	n.d.	n.d.	n.d.
5,8,11,14,17-Eicosapentaenoic acid	C20:5	0.82 ± 0.19[ab]	0.91 ± 0.09[a]	0.89 ± 0.08[a]	0.65 ± 0.13[cd]	0.72 ± 0.07[bc]	0.54 ± 0.04[de]	0.51 ± 0.08[e]
cis-13,16-Docasadienoic acid	C22:2	n.d.	n.d.	n.d.	n.d.	n.d.	n.d.	n.d.
4,7,10,13,16,19-Docosahexaenoic acid	C22:6	n.d.	n.d.	n.d.	n.d.	n.d.	n.d.	n.d.
Total SFAs		*534.9 ± 119.7*	*675.5 ± 76.6*	*641.6 ± 57.8*	*487.5 ± 96*	*510.2 ± 45.2*	*391 ± 28.3*	*369.6 ± 47.9*
Total MUFAs		*164.4 ± 37*	*225.8 ± 32.1*	*228.5 ± 16.4*	*176 ± 27.9*	*177.4 ± 14.7*	*137.6 ± 11.4*	*135.8 ± 19*
Total PUFAs		*13.8 ± 3.2*	*71.9 ± 10.7*	*119.4 ± 9.8*	*96.9 ± 21.2*	*46 ± 3.5*	*61.1 ± 5.1*	*73.1 ± 10.9*
% SFAs		*75.01*	*69.41*	*64.84*	*64.11*	*69.55*	*66.30*	*63.89*
% MUFAs		*23.05*	*23.20*	*23.10*	*23.14*	*24.18*	*23.33*	*23.47*
% PUFAs		*1.94*	*7.38*	*12.07*	*12.75*	*6.27*	*10.36*	*12.63*
Atherogenic index (AI)		*3.58*	*2.74*	*2.23*	*2.09*	*2.77*	*2.32*	*2.09*
Thrombogenic index (TI)		*3.31*	*2.87*	*2.42*	*1.95*	*2.60*	*2.04*	*1.74*

Saturation index (SI)	*2.11*	*1.60*	*1.29*	*1.19*	*1.59*	*1.35*	*1.22*

* n.d.: not detected. Data are presented as Mean ± SD (n = 3) and listed in the order of group and then number of carbons. Different alphabets superscripts indicate statistical difference ($p < 0.05$) across each row
